# Investigation of long non-coding RNAs as regulatory players of grapevine response to powdery and downy mildew infection

**DOI:** 10.1186/s12870-021-03059-6

**Published:** 2021-06-08

**Authors:** Garima Bhatia, Santosh K. Upadhyay, Anuradha Upadhyay, Kashmir Singh

**Affiliations:** 1 Department of Biotechnology, Panjab University, BMS Block I, Sector 25, Chandigarh, 160014 India; 2 Department of Botany, Panjab University, Chandigarh, 160014 India; 3 National Research Centre for Grapes, Solapur Road, Pune, Maharashtra 412307 India

**Keywords:** *Vitis vinifera*, Powdery mildew, Downy mildew, LncRNAs, MiRNAs, Transcription factors, Defense response

## Abstract

**Background:**

Long non-coding RNAs (lncRNAs) are regulatory transcripts of length > 200 nt. Owing to the rapidly progressing RNA-sequencing technologies, lncRNAs are emerging as considerable nodes in the plant antifungal defense networks. Therefore, we investigated their role in *Vitis vinifera* (grapevine) in response to obligate biotrophic fungal phytopathogens, *Erysiphe necator* (powdery mildew, PM) and *Plasmopara viticola* (downy mildew, DM), which impose huge agro-economic burden on grape-growers worldwide.

**Results:**

Using computational approach based on RNA-seq data, 71 PM- and 83 DM-responsive *V. vinifera* lncRNAs were identified and comprehensively examined for their putative functional roles in plant defense response. *V. vinifera* protein coding sequences (CDS) were also profiled based on expression levels, and 1037 PM-responsive and 670 DM-responsive CDS were identified. Next, co-expression analysis-based functional annotation revealed their association with gene ontology (GO) terms for ‘response to stress’, ‘response to biotic stimulus’, ‘immune system process’, etc. Further investigation based on analysis of domains, enzyme classification, pathways enrichment, transcription factors (TFs), interactions with microRNAs (miRNAs), and real-time quantitative PCR of lncRNAs and co-expressing CDS pairs suggested their involvement in modulation of basal and specific defense responses such as: Ca^2+^-dependent signaling, cell wall reinforcement, reactive oxygen species metabolism, pathogenesis related proteins accumulation, phytohormonal signal transduction, and secondary metabolism.

**Conclusions:**

Overall, the identified lncRNAs provide insights into the underlying intricacy of grapevine transcriptional reprogramming/post-transcriptional regulation to delay or seize the living cell-dependent pathogen growth. Therefore, in addition to defense-responsive genes such as TFs, the identified lncRNAs can be further examined and leveraged to candidates for biotechnological improvement/breeding to enhance fungal stress resistance in this susceptible fruit crop of economic and nutritional importance.

**Supplementary Information:**

The online version contains supplementary material available at 10.1186/s12870-021-03059-6.

## Background

Long non-coding RNAs (lncRNAs) are transcripts longer than 200 nt but lacking known coding potential, which along with other regulatory RNAs help in coordinating biological processes across eukaryotes. In plants, besides regulating developmental transitions and reproduction, they have been associated with response to stress conditions (reviewed in [1, 2]). Although the initial pace of lncRNAs research in plants was slower compared to that in mammals (especially humans), it has gained momentum in the last few years with the advancement in high-throughput sequencing technologies and the availability of genomic and transcriptomic information of several plants at high resolution.

One such plant is *Vitis vinifera* (grapevine), which has been extensively studied owing to its commercial importance and worldwide consumption. It encompasses nearly 5000 cultivars that are used widely for both fresh and dried grape consumption and wine production [3, 4]. However, this economically important fruit crop is affected by abiotic and biotic stress conditions [5, 6]. It is susceptible to many pathogens and pests; of which, fungal and oomycetes phytopathogens pose grave risks during different phases of production. Particularly, powdery and downy mildew (PM and DM) diseases caused by obligate biotrophic fungus *Erysiphe necator* and oomycete *Plasmopara viticola*, respectively, have been associated with economic losses worldwide [7]. To avoid these losses, chemical treatments like fungicides have been largely applied in viticulture, which are costly not only for crop growers but also the environment [7, 8]. Therefore, efforts are being made to understand the underlying mechanisms of *V. vinifera* susceptibility to PM and DM, and in turn engineer the cultivated grapevine for resistance against these phytopathogens [4, 7, 8].

In this direction, studies have been conducted to analyze plant defense response at transcript, protein, and metabolite levels [8–14]. Also, micro RNAs (miRNAs) have been identified in response to *E. necator* in resistant Chinese wild species, *Vitis pseudoreticulata* [15]. However, the regulation of *V. vinifera* response to PM and DM with respect to lncRNAs has not been explored till date. Previously, in independent studies, *V. vinifera* lncRNAs have been identified as potential regulators at different developmental stages, in response to cold stress, and upon infection with hemibiotrophic and necrotrophic fungal pathogens *Lasiodiplodia theobromae* and *Botrytis cinerea*, respectively [16–19]. Unlike necrotrophs and hemibiotrophs that eventually favor plant cell death for nourishment, obligate biotrophic phytopathogens such as *E. necator* and *P. viticola* sustain exclusively on living *V. vinifera* cells. Therefore, it would be interesting to explore their role in regulation of plant defense response that is dependent on extensive transcriptional reprogramming.

With this background, we harnessed 56,441 *V. vinifera* lncRNAs (previously identified by our lab) to investigate their response to *E. necator* and *P. viticola* infection. We found 71 and 83 PM- and DM-responsive lncRNAs, respectively, which have provided us fresh insights into the regulation of plant response against biotrophic pathogens. In addition to defense-responsive genes such as transcription factors, the identified lncRNAs can be further examined and leveraged to candidates for biotechnological improvement/breeding to enhance fungal stress resistance in this perennial fruit crop*.*

## Results

### Genome-wide identification of powdery and downy mildew-responsive lncRNAs in *V. vinifera*

**Fig. 1:**
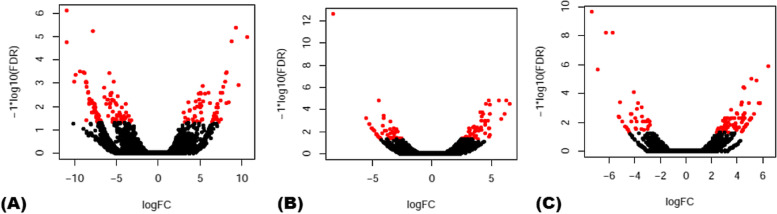
Volcano plots for visualization of pair-wise comparison of lncRNAs expression profile between samples: **A** control versus *Erysiphe necator* (powdery mildew) infection at 36 hpi **B** control versus *Plasmopara viticola* (downy mildew) infection at 24 hpi and **C** 48 hpi. The x-axis corresponds to log2 (fold change) between the samples (that is, logFC), and the y-axis corresponds to false discovery rate (that is, − log_10_FDR). LncRNAs identified as significantly differentially expressed are shown in red color

Differential expression analysis of 56,441 V*. vinfera* lncRNAs based on different biotic stress conditions (Additional File 1: Table S1) revealed 71 PM- and 83 DM-responsive lncRNAs (*P*-values [FDR] <  = 0.01 and fourfold change) (Fig. 1; Additional File 2: Figure S1). Similar analysis for 37,420 V*. vinifera* CDS revealed 1037 PM- and 670 DM-responsive protein coding transcripts (Additional File 2: Figure S2). Further, it was observed that many of the deregulated lncRNAs (60.6%) showed an up-regulation in response to PM infection. A similar trend was observed for the PM-responsive CDS as 63.4% transcripts were up-regulated under this biotic stress. However, more than half (nearly 67.5%) lncRNAs were observed to be down-regulated in response to DM infection. The DM-responsive CDS exhibited similar expression trends, that is, nearly 65.1% transcripts were down-regulated. Of the identified PM- and DM-responsive lncRNAs in the plant, only one, that is, TR78139, was found to be common in response to both the obligate biotrophic pathogens (Additional File 2: Figure S3).

### Functional annotation of the identified biotic stress-responsive *V. vinifera* lncRNAs

To gain insights into the plausible roles of the identified PM- and DM- responsive *V. vinifera* lncRNAs, their functional annotation was conducted. This was based on their co-expression with the differentially expressing CDS in response to the two obligate biotrophic pathogens. Co-expression correlation between the two transcript categories was calculated using Pearson correlation coefficient with *R* >  = 0.9 (Additional File 2: Figure S4; Additional File 3). The highly correlated pairs were identified (*R* >  = 0.9), and it was found that 52 PM-responsive lncRNAs co-expressed with 33 CDS. Out of the 83 differentially expressing DM-responsive lncRNAs, 29 could be filtered out for *P-*value (cut off for FDR) <  = 0.001 and were used for co-expression analysis as high-confidence candidates. Consequently, 22 DM-responsive lncRNAs were observed to co-express with 127 CDS. The networks representing potential interactions between lncRNAs and CDS pairs based on co-expression have been included in Additional File 2: Figures S5-S7. It was observed that many lncRNAs could potentially be associated with a CDS and vice-versa.

- Gene ontology (GO) enrichment analysis

GO enrichment analysis was performed for all the aforementioned co-expressing CDS using Blast2GO tool [20]. As a part of this analysis, hits were obtained by BLAST search for all the PM-responsive sequences, and 90.91% of these were mapped against different database sources such as UniProt, EnsemblPlants, TAIR, etc. for retrieval of GO terms. Accordingly, 81.81% of the sequences were functionally annotated with at least one GO term in the following three categories: cellular component (CC), molecular functions (MF) and biological processes (BP) (Additional File 4). For instance, (i) in CC category, we observed GO terms such as GO:0,044,464 ‘cell part’, GO:0,005,576 ‘extracellular region’, and GO:0,016,020 ‘membrane’. (ii) In MF category, GO terms such as GO:0,003,824 ‘catalytic activity’, GO:0,005,488 ‘binding’, and GO:0,140,110 ‘transcription regulator activity’ were found. (iii) Finally, in BP category, GO terms such as GO:0,065,007 ‘biological regulation’, GO:0,050,896 ‘response to stimulus’, GO:0,050,789 ‘regulation of biological process’ and GO:0,002,376 ‘immune system process’ were observed. Overall, the results indicate putative lncRNA involvement in ‘regulation’ of plant response to PM. Additionally, direct GO count for BP category was analyzed, which represents the most frequent GO terms within the data-set excluding GO hierarchy (Additional File 4). Terms corresponding to ‘oxidation–reduction process’, ‘regulation of transcription, DNA-templated’, ‘response to hydrogen peroxide’, ‘proteolysis’, and ‘cell wall organization’ were observed, which highlight putative role of lncRNAs in plant basal defense response against the invading fungal pathogen.

Similarly, BLAST hits were obtained for all the co-expressing DM-responsive CDS. Of which, 98.4% could be mapped and 94.48% could be assigned at least one GO term in the above-mentioned three categories (Additional File 4). For instance, (i) the CC category included terms such as GO:0,005,622 ‘intracellular’, GO:0,043,227 ‘membrane-bounded organelle’ and GO:0,071,944 ‘cell periphery’. (ii) The MF category included terms such as GO:0,043,167 ‘ion binding’, GO:0,016,491 ‘oxidoreductase activity’ and GO:0,003,700 ‘DNA binding transcription factor activity’. (iii) The BP category included terms like GO:0,006,950 ‘response to stress’, GO:0,009,607 ‘response to biotic stimulus’ and GO:0,009,605 ‘response to external stimulus’. Like PM-responsive lncRNAs, direct GO count for BP category indicated involvement of DM-responsive lncRNAs in processes like- ‘oxidation–reduction process’, ‘regulation of transcription, DNA-templated’, ‘cell wall organization’, etc. Moreover, many terms such as ‘response to chitin’, ‘defense response to fungus’, ‘response to oomycetes’, ‘killing of cells of other organism’, ‘positive regulation of cell death’, ‘defense response by callose deposition in cell wall’ etc. were observed, which highlight the potential involvement of DM-responsive lncRNAs in mediating defense-oriented transcriptional programming associated with post-infection plant defense responses (Additional File 4).

**Fig. 2:**
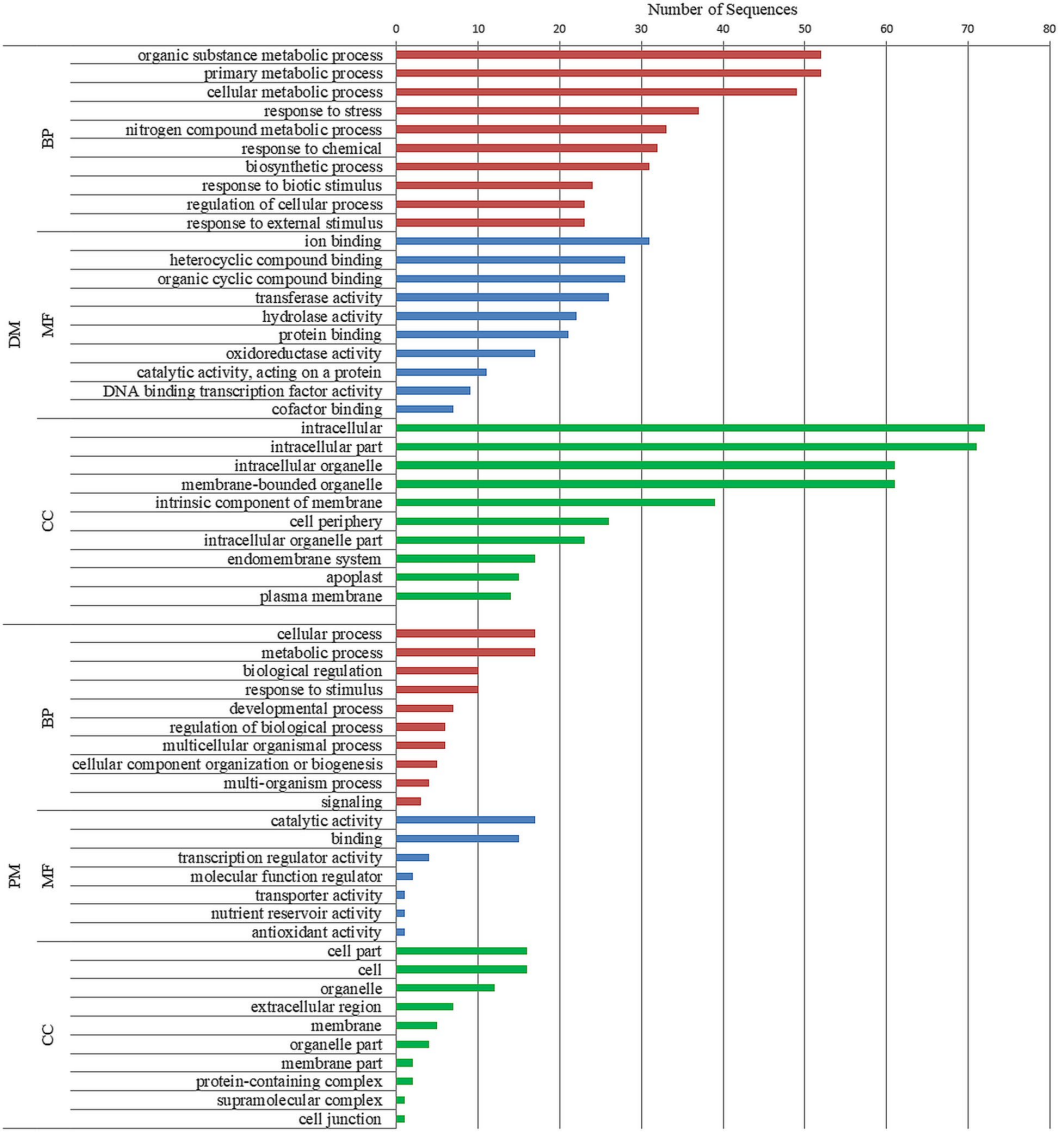
Top gene ontology (GO) Terms showing enrichment for PM- and DM-responsive lncRNAs co-expressing with protein coding sequences: The enrichment is represented in three categories: BP, biological process; MF, molecular function; and CC, cellular component. PM, powdery mildew (*Erysiphe necator*); DM, downy mildew (*Plasmopara viticola*)

Broadly, Fig. 2 shows the top ten terms for all the three categories (taking into account GO hierarchy) suggesting the possible functions these identified lncRNAs could be playing in the plant in response to PM and DM.

- Domain analysis

**Fig. 3:**
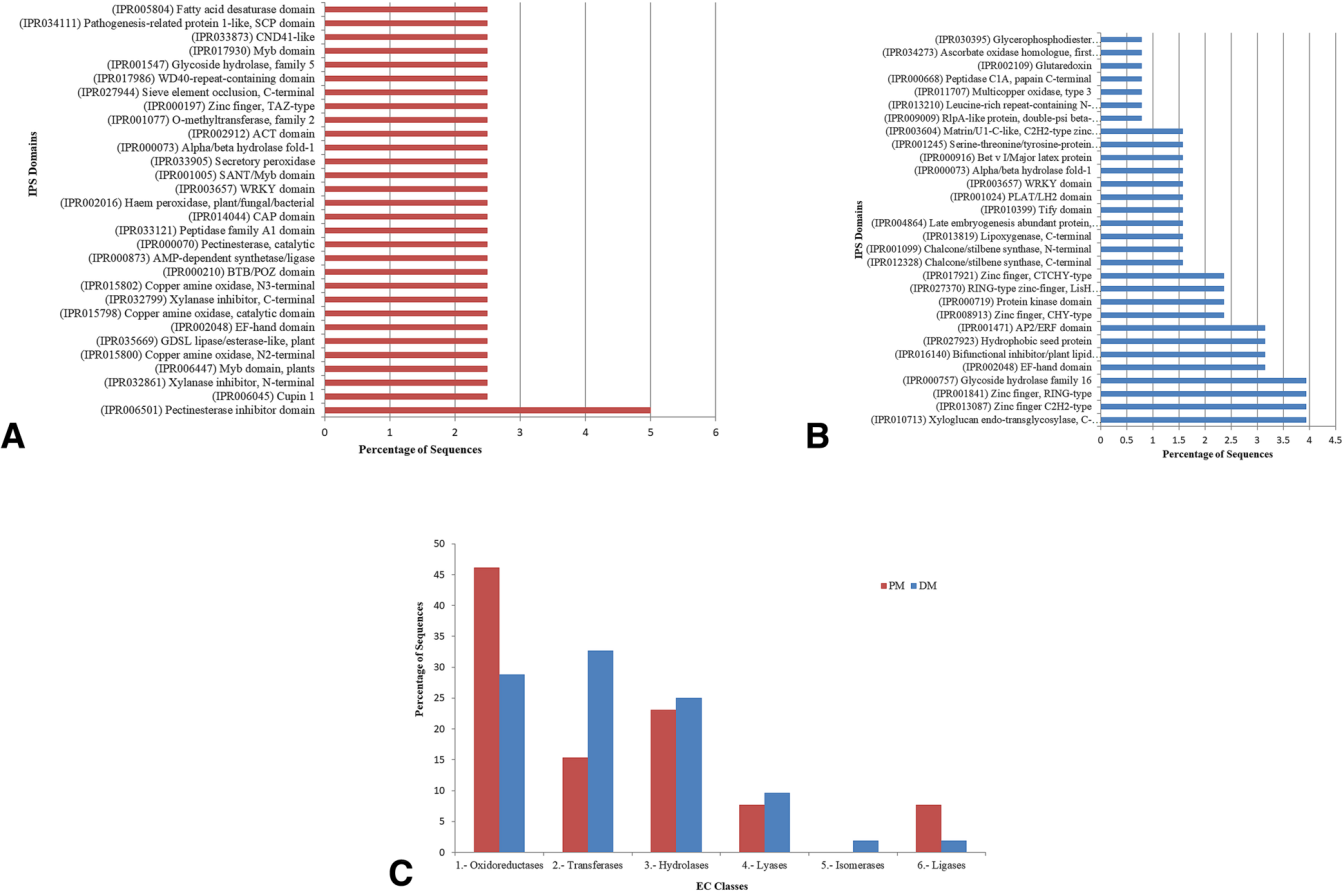
Domain- and enzyme-based functional annotation: Domain distribution of co-expressing protein coding sequences with **A** PM-responsive and **B** DM-responsive lncRNAs into different categories as per GO terms. **C** Enzyme code classification of protein coding sequences with the identified lncRNAs. PM, powdery mildew (*Erysiphe necator*); DM, downy mildew (*Plasmopara viticola*)

Next, as a part of functional analysis of PM- and DM- responsive lncRNAs, we conducted domain analysis for the co-expressing CDS using InterProScan. The predicted domains and sites provided further insight into the potential involvement of lncRNAs in response to biotic stress (Fig. 3A, B; Additional File 2: Figure S8). For instance, domains such as pectinesterase inhibitor domain (IPR006501), xylanase inhibitor C-terminal (IPR032799), secretory peroxidase (IPR033905) and copper amine oxidase N2-terminal (IPR015800) indicate the plausible involvement of co-expressing-lncRNAs in regulating changes in the redox state of cells and cell wall reinforcement as basal defense response against PM infection (Fig. 3A).

In the case of DM-responsive co-expressing lncRNAs-CDS pairs, the predicted domains were associated with defense responses (Fig. 3B) such as, (i) cell wall modification: xyloglucan endo-transglycosylase C-terminal (IPR010713), glycoside hydrolase family 16 (IPR000757); (ii) phytoalexin production: chalcone/stilbene synthase C-terminal and N-terminal (IPR012328, IPR001099); (iii) pathogenesis-related proteins: PR-10, Bet v I/Major latex protein (IPR000916); (iv) DNA/RNA/protein binding: zinc finger C2H2-type and RING-type (IPR013087, IPR001841); (v) protein kinases: serine-threonine/tyrosine-protein kinase catalytic domain (IPR001245); and (vi) others like: leucine-rich repeat-containing N-terminal plant-type (IPR013210).

Additionally, some common domains were found indicating parallel plant defense responses against both the biotrophic pathogens. These included- hydrolases ‘GDSL lipase/esterase-like plant’ (IPR035669), calcium-binding ‘EF-hand domain’ (IPR002048), regulatory WRKY domain (IPR003657), pathogenesis-related protein 1-like, SCP domain (IPR034111) and/or cysteine-rich secretory proteins CAP domain (IPR014044).

- Enzyme code based classification

The annotated coding sequences co-expressing with PM- and DM- responsive lncRNAs were further classified based on enzyme codes (EC) and their distribution patterns were studied (Fig. 3C). Out of the six major EC classes, the maximum co-expressing lncRNA-CDS pairs belonged to oxidoreductases, transferases, and hydrolases classes in response to both the biotrophic phytopathogens.

- Pathways enrichment analysis

**Fig. 4:**
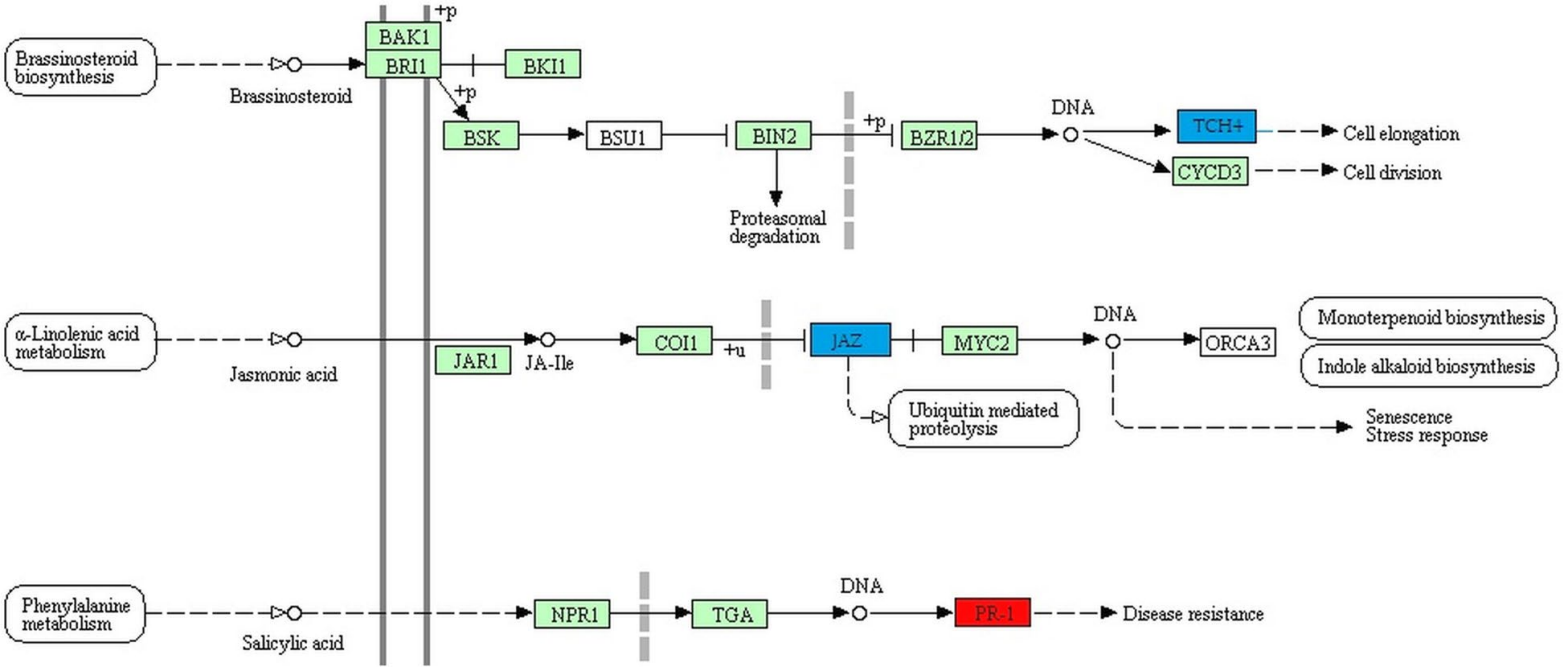
Pathways enrichment analysis: Potential involvement of lncRNAs (co-expressing with protein coding sequences) in ‘plant hormone signal transduction’ pathway in powdery mildew and downy mildew infections highlighted in red and blue colors, respectively

Pathways enrichment analysis was conducted based on KEGG pathways database specifically for *V. vinifera* [21], and results suggested potential involvement of the identified fungal and oomycete stress-responsive lncRNAs in representatives of 39 pathways (Additional File 5, Additional File 2: Figure S9). Ten pathways were exclusively enriched in response to PM; for instance, ‘glycine, serine and threonine metabolism’, ‘isoquinoline alkaloid biosynthesis’, ‘phenylalanine metabolism’, and ‘phenylpropanoid biosynthesis’. In response to DM, exclusive enrichment was observed for 21 pathways including ‘alpha-linolenic acid metabolism’, ‘stilbenoid, diarylheptanoid and gingerol biosynthesis’, ‘flavonoid biosynthesis’, and ‘diterpenoid biosynthesis’. Interestingly, 8 common pathways were observed in response to both the biotrophic phytopathogens, which included ‘metabolic pathways’, ‘biosynthesis of secondary metabolites’, ‘plant-pathogen interaction’ and ‘plant hormone signal transduction’. However, the co-expressing lncRNA-CDS pairs were different for these pathways in the two stress conditions. The differences can be seen for ‘plant hormone signal transduction’ pathway in Fig. 4 and for ‘plant-pathogen interaction’ pathway in Additional File 2: Figure S10.

### Co-expressing lncRNAs and transcription factor pairs in response to powdery and downy mildew infections

**Fig. 5:**
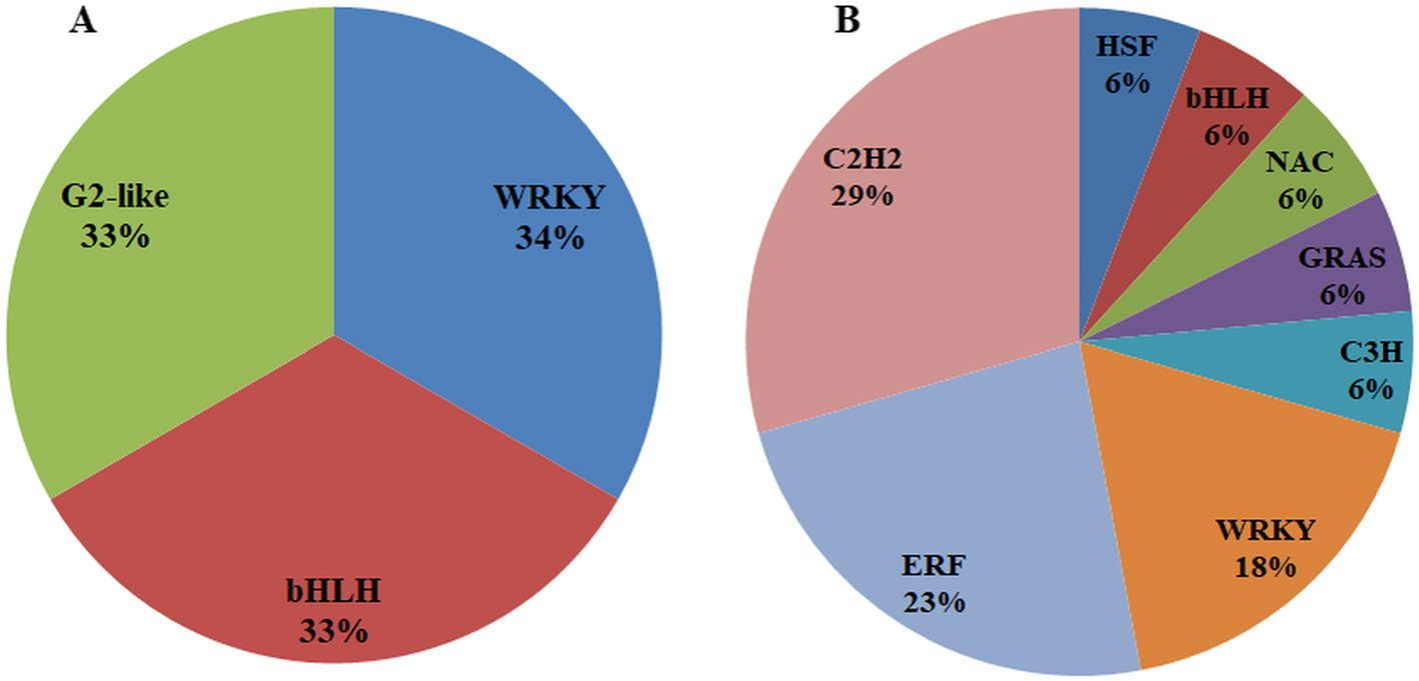
Transcription factor (TF) families co-expressing with PM- and DM-responsive *Vitis vinifera* lncRNAs. WRKY and bHLH TF families were found common. PM, powdery mildew (*Erysiphe necator*); DM, downy mildew (*Plasmopara viticola*)

During functional annotation analysis, we observed that the identified lncRNAs were potentially associated with transcriptional regulation (based on GO terms) and domain analysis also revealed DNA/RNA/protein binding domains. Therefore, we further investigated the potential association of lncRNAs with transcription factors (TF). TF families co-expressing with PM- and DM-responsive *V. vinifera* lncRNAs were identified using prediction server based on Plant TF database v5.0 (Fig. 5). Three co-expressing lncRNA-TF pairs corresponding to WRKY, bHLH, and G2-like were observed in response to PM. In response to DM, 17 lncRNAs co-expressed with 8 TF families including stress-responsive C2H2, ERF, HSF, GRAS, C3H and NAC. WRKY and bHLH were common in response to both the biotrophic pathogens.

### Interaction analysis of powdery and downy mildew-responsive *V. vinifera* lncRNAs with miRNAs

**Fig. 6:**
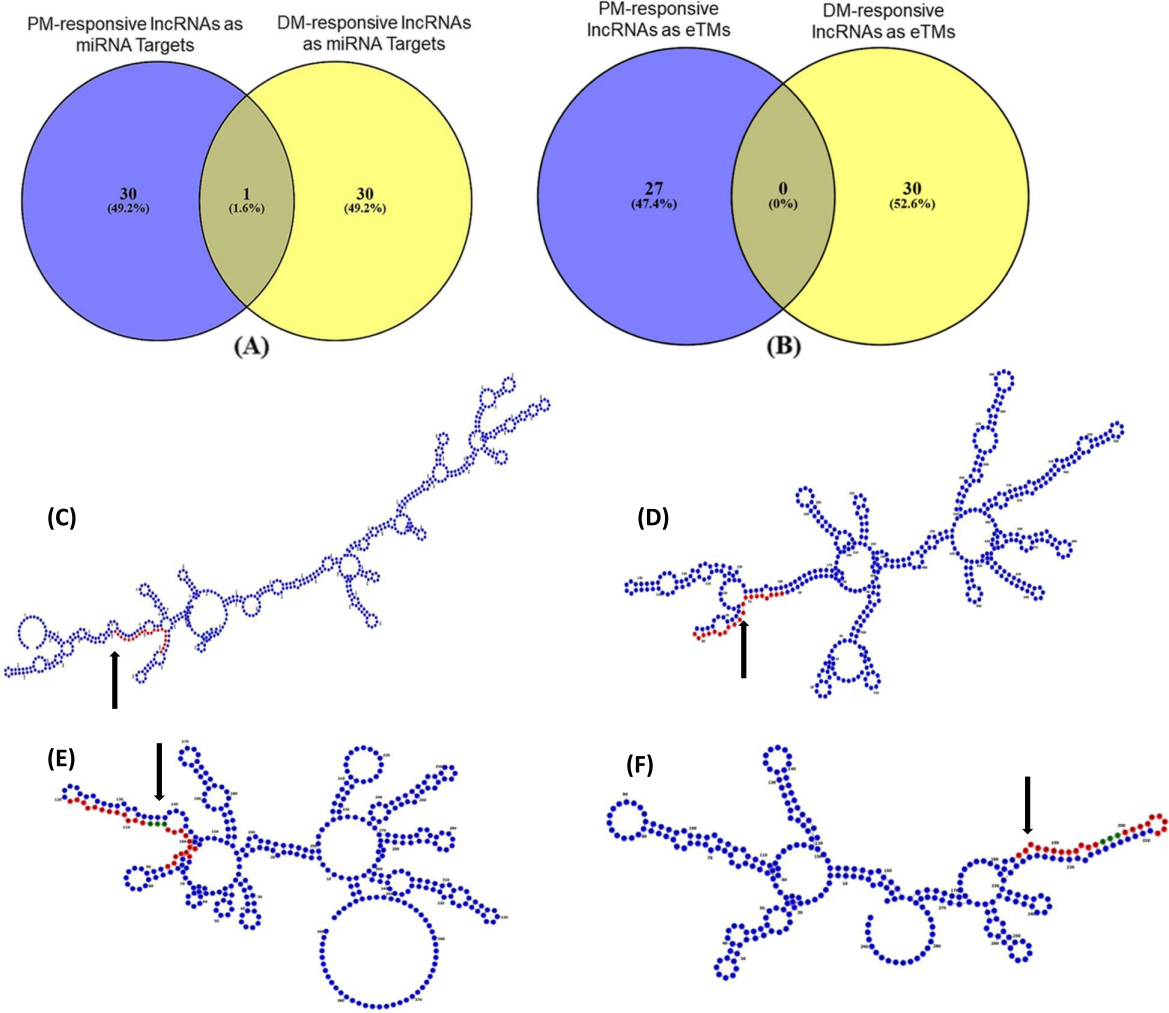
PM- and DM-responsive lncRNAs as putative targets and endogenous target mimics (eTMs) of *Vitis vinifera* miRNAs. **A** A Venn diagram showing PM- and DM-resposive lncRNAs that can act as targets of vvi-miRNAs. **B** A Venn diagram showing PM- and DM-resposive lncRNAs that can act as endogenous target mimics of vvi-miRNAs. **C** Secondary structure of a PM-responsive lncRNA (TR36037) shown in blue, which acts as a putative target of miRNA (vvi-miR164d) shown in red. **D** Secondary structure of a DM-responsive lncRNA (TR229744) shown in blue, which acts as a putative target of miRNA (vvi-miR156h) shown in red. **E** Secondary structure of a PM-responsive lncRNA (TR63892) shown in blue, which acts as a putative eTM for miRNA (vvi-miR172c) shown in red. The characteristic 3-nt bulge is shown in green. **F** Secondary structure of a DM-responsive lncRNA (TR55735) shown in blue, which acts as a putative eTM for miRNA (vvi-miR172c) shown in red. The characteristic 3-nt bulge is shown in green

**Fig. 7:**
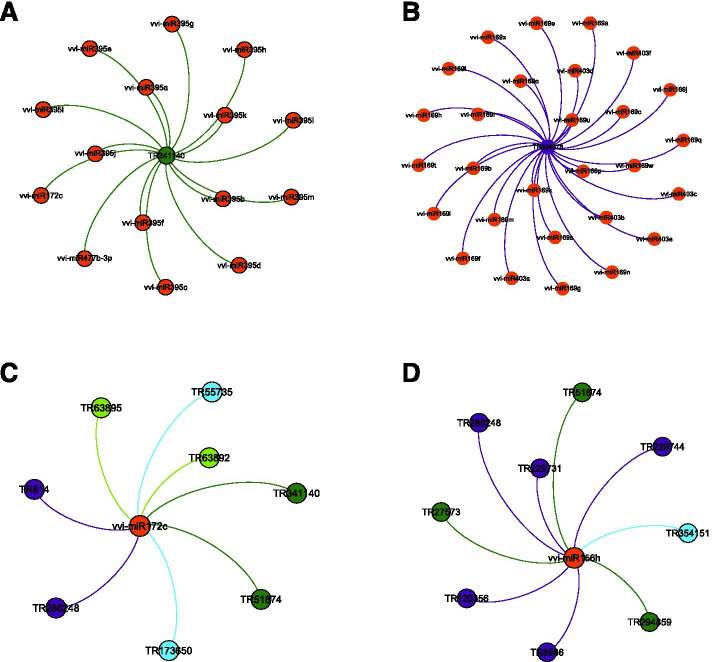
Interaction network analysis representing **A** Interaction of a PM-responsive lncRNA (green) with multiple miRNAs (red). **B** Interaction of a DM-responsive lncRNA (blue) with multiple miRNAs (red). **C** and **D** Interaction of an miRNA (red) with multiple lncRNAs (PM-responsive, green; DM-responsive, blue). A potential eTM is marked in cyan

To gain perspective on regulatory relationships between short and long ncRNAs in response to biotrophic pathogen attack, the identified lncRNAs were examined for the presence of target sites of *V. vinifera*-specific mature miRNAs (Fig. 6A). We found 31 PM- and 31 DM-responsive lncRNAs that could act as potential targets of 78 and 105 miRNAs, respectively (Additional File 6, Additional File 2: Figure S11). Of these, only one lncRNA was common in the two conditions; however, 52 common miRNAs targeting lncRNAs were observed. Next, lncRNAs that could act as endogenous target mimics (eTMs) for miRNAs were determined for the two biotic stress conditions (Fig. 6B). We identified 27 PM- and 30 DM-responsive lncRNAs as putative eTMs for 30 and 35 miRNAs, respectively (Additional File 6, Additional File 2: Figure S12). While no lncRNAs as putative eTMs were found common in response to PM and DM, 19 of the associated miRNAs were found common. The identified lncRNAs potentially interact with miRNAs, which have been studied in response to biotic stress conditions, for instance, miR156, miR159, miR164, miR172, miR319, miR396 and miR482. Additionally, we found that 49 and 42 miRNAs, for which DM-responsive lncRNAs can act as targets and target mimics respectively, also target correlated and co-expressing DM-responsive mRNAs. Moreover, 11 and 12 miRNAs, for which PM-responsive lncRNAs can act as targets and target mimics respectively, also target correlated and co-expressing PM-responsive mRNAs (Additional File 6). Figures 6C-F represent examples of secondary structure prediction of PM- and DM-responsive lncRNAs as putative targets and eTMs of *V. vinifera* miRNAs. Finally, the interaction analyses of the PM- and DM-responsive lncRNAs with *V. vinifera* miRNAs were visualized to gain an overview of the interactomes (Fig. 7, Additional File 7).

### qRT-PCR of select biotic stress-responsive lncRNAs and co-expressing protein coding sequences

**Fig. 8:**
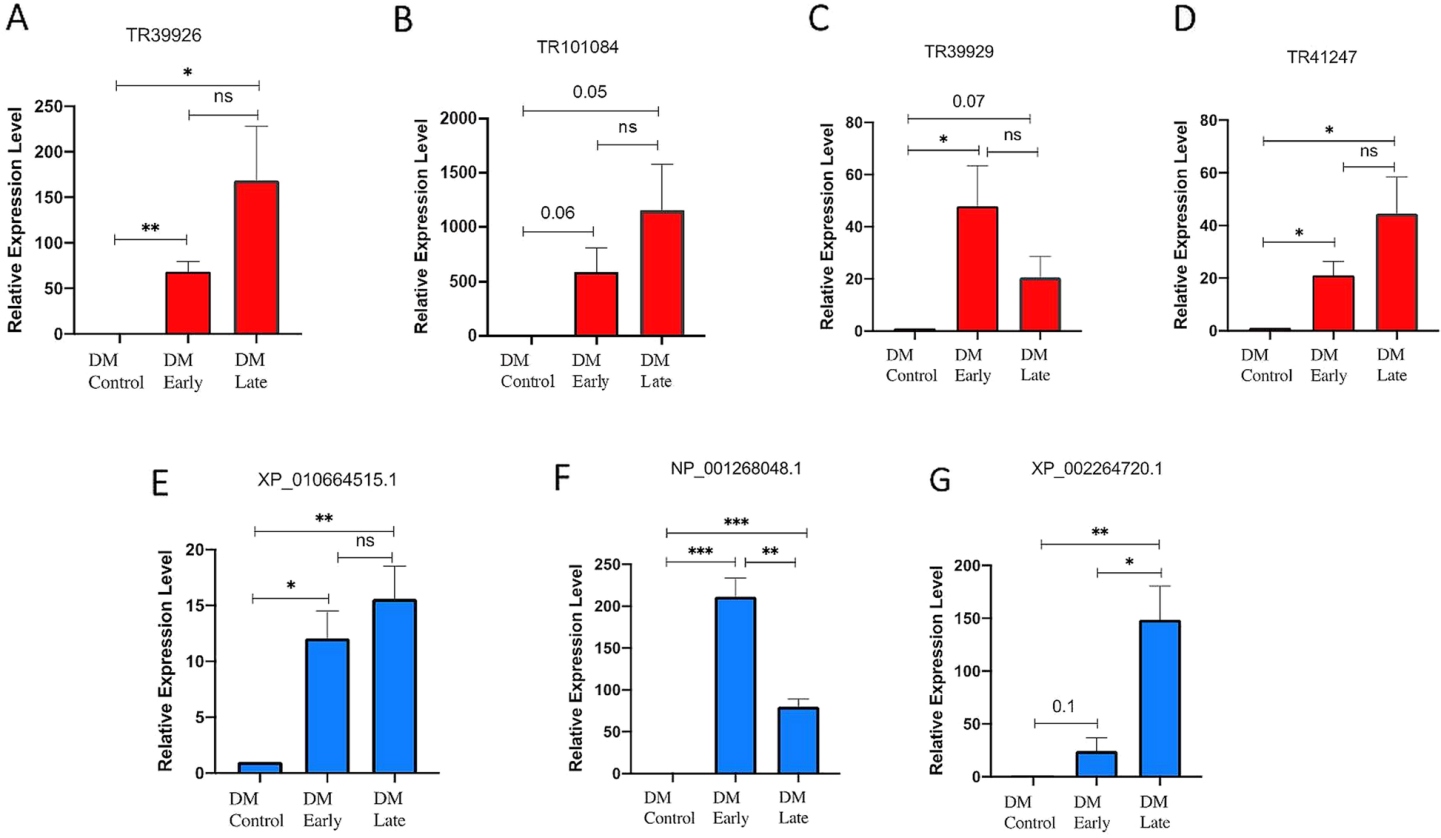
Relative expression analysis of select downy mildew (DM)-responsive lncRNAs (**A**-**D**) and protein coding sequences (**E**–**F**) using quantitative real time polymerase reaction. Both the selected lncRNAs and protein CDS exhibit up-regulation in response to DM infection (as depicted by red and blue columns, respectively). *Actin-7 (Act-7)* was used for normalization of gene expression. Early and late infection correspond to 1 and 3 dpi, respectively. Results are depicted as mean ± SE of the triplicates. Statistical analysis has been performed by using unpaired *t-*test. *, **, and *** indicate significance as *p* ≤ 0.05, *p* ≤ 0.01, and *p* ≤ 0.001, respectively. ns, not significant or *p* > 0.05; dpi, days post inoculation

Of the differentially expressed biotic stress-responsive lncRNAs identified in this study, high-confidence lncRNAs in response to DM (*P-*value [FDR] <  = 0.001, fourfold change, FPKM > 100) were considered for qRT-PCR analysis. Of these, some candidates were randomly selected for validating their differential expression profile. Consequently, similar trends of expression were observed as those seen based on RNA-seq data, especially for the common time-point of infection, that is, 24 hpi or 1 dpi (Additional File 2: Figure S13). LncRNAs TR39926, TR39929, TR41247, and TR101084 were observed to be up-regulated in response to DM (Fig. 8). Moreover, lncRNAs TR39926 and TR101084 were found to be up-regulated at both early (1 dpi) and advanced (3 dpi) stages of DM infection (Fig. 8A, B). Interestingly, lncRNAs TR39929 and TR41247 exhibited similar trends of expression upon DM infection, that is up-regulation, as XP_002264720.1, which is the coding sequence for pathogenesis-related protein (PR)-4 (Fig. 8C, D, and G). Additionally, lncRNA TR39929 was found to co-express with NP_001268048.1, which is the coding sequence for another defense-responsive protein- acidic endochitinase precursor (Fig. 8C and F). Likewise, both lncRNA TR101084 and XP_010664515.1 (coding for probable strigolactone esterase DAD2) were found to be up-regulated in response to DM infection. The co-expression patterns of these selected DM-responsive lncRNAs and their corresponding defense-responsive protein CDS determined by both in silico differential expression analysis (FPKM values) and qRT-PCR-based analysis were found to overlap upon DM infection and have been depicted in the Additional File 2: Figure S14.

## Discussion

Owing to its susceptibility to diseases such as those caused by fungal and oomycete phytopathogens, *V. vinifera* exhibits defense responses that are driven by extensive changes at the transcriptional level. Particularly, in response to biotrophic fungal phytopathogen *E. necator*, the disease-susceptible *V. vinifera* has been reported to undergo a greater extent of transcriptional reprogramming compared to its disease-resistant counterpart, *Vitis aestivalis* [9]. In contrast, drastic transcriptional-level changes have been reported in both disease-resistant (*V. riparia*) and –susceptible (*V. vinifera*) grapevine species in response to the biotrophic oomycete *P. viticola*. In fact, the resistant plant demonstrates a faster and stronger defense-oriented transcriptional remodeling and a milder version of the response against DM is observed in susceptible grapevine [22]. In either case, the importance of understanding the underlying regulation of transcriptional changes upon intrusion of biotrophic pathogens cannot be undermined.

Despite themselves being products of transcription, lncRNAs have emerged as major regulators of the process. Additionally, these transcripts can mediate regulation at post-transcriptional and post-translational levels [23, 24]. In view of their regulatory versatility, lncRNAs can be leveraged as candidates for biotechnological improvement of crops in addition to the conventional approach of over-expressing defense-related genes or transcription factors (TFs) [7, 24]. Till date, efforts to understand lncRNA-mediated plant response to obligate biotrophic fungal pathogens have been primarily conducted in *Triticum aestivum* (wheat), against *Blumeria graminis* f. sp. *tritici* and *Puccinia striiformis* f. sp. *tritici,* which cause PM and stripe rust disease in the plant, respectively [25, 26]. To our knowledge, this is the first study based on genome-wide investigation of lncRNA-mediated response to obligate biotrophic pathogens causing PM and DM in susceptible grapevine. We identified 71 and 83 PM- and DM-responsive lncRNAs in *V. vinifera*; of which, only one lncRNA was common. This observation was not unexpected because lncRNAs are known to express at specific sites (tissues/cells) and in response to specific conditions/stimuli [24, 27]. In contrast, 94 differentially expressed CDS were found common in response to the two biotrophic pathogens. However, it is important to mention here that this observation could also be an outcome of the differences in the plant materials and infection time-points in the two independent studies, which were the source of transcriptomic data for the current study (as mentioned in Additional File 1: Table S1). Moreover, our study harnesses 56,441 V*. vinifera*-specific lncRNAs, which were identified across different tissues and development stages using a de novo approach [16]. Therefore, there is scope for further studies based on novel sequencing strategies to identify additional lncRNAs in response to infection.

Next, expression profiling revealed that a majority of the responsive CDS was up-regulated in response to PM and down-regulated in response to DM as also observed in earlier studies on the susceptible grapevine plant [9, 28]. Interestingly, similar expression profiles were observed for PM- and DM-responsive lncRNAs identified in this study. Subsequently, these differentially expressing lncRNAs were functionally annotated based on their co-expression with CDS, and the results indicated their association with biological processes involved in response to biotrophic stress. Despite their phylogenetic distance, true fungi and oomycetes, as biotrophic pytopathogens, share certain features that help them successfully invade (by development of appresoria, infection hyphae, and haustoria) and sustain within the host plant’s living cells [29]. Hence, the host plant’s responses to the two obligate biotrophs are likely to overlap. In the present study, domain analysis of the co-expressing CDS with respect to PM- and DM-responsive lncRNAs revealed some common domains associated with plant responses such as lipid metabolism [30], calcium ion binding protein-mediated signaling [31], WRKY gene family-mediated transcriptional regulation [32], and pathogenesis-related protein 1 (PR-1) accumulation [33]. This indicates the possible involvement of the identified *V. vinifera* lncRNAs in regulating some common defense responses against biotrophs. Recently, ELF18-INDUCED LONG-NONCODING RNA1 (ELENA1) was identified as a positive regulator of plant resistance to *Pseudomonas syringe* pv. tomato DC3000 (hemi-biotrophic bacteria) based on increased *PR*-*1* expression observed in *Arabidopsis thaliana* [34]. In another recent study on *Solanum lycopersicum* (tomato), lncRNA33732 was found to be activated by WRKY1, which in turn enhanced early defense response against *Phytophthora infestans* (hemi-biotrophic oomycete) [35]. Furthermore in the present study, particularly in response to PM, CDS with domains associated with inhibitors of cell-wall degrading enzymes (CWDE), such as pectinesterase and xylanase, were observed to co-express with lncRNAs. This suggests that PM-responsive lncRNAs could potentially regulate the inhibition of secreted fungal CWDE to avoid cell wall damage caused by fungi while invading plant tissue for haustoria development, thereby, helping to maintain plant cell wall integrity [8, 29, 36, 37]. Additionally, PM-responsive lncRNAs were associated with domains such as copper amine oxidase, which generates reactive oxygen species (ROS) like hydrogen peroxide (H_2_O_2_) upon amine degradation [38], and has been associated with events such as oxidative burst, cell death, and peroxidase-mediated lignification during biotic stress [39]. Likewise, DM-responsive lncRNAs were found to co-express with lipoxygenase domains that have been associated with stress-induced hydroperoxidation of polyunsaturated fatty acids leading to ROS production [40]. This indicates the potential involvement of PM- and DM-responsive lncRNAs in regulating ROS-mediated defense response in *V. vinifera*. Previously, lncRNA-mediated induction of ROS scavenger glutaredoxin has also been reported in tomato in response to *P. infestans* [41].

The identified DM-responsive lncRNAs were also found to co-express with CDS including domains for chalcone/stilbene synthase, which indicates the potential regulation of secondary metabolism and phytoalexin (like resveratrol) production against the pathogen in the susceptible grapevine plant [13, 42]. Moreover, domains associated with defense-related PR-10 proteins, which possess nuclease activity, highlight the potential involvement of co-expressing DM-responsive lncRNAs in dual regulation of pathogenic RNA degradation and host programmed cell death [43].

To further delve into the putative functions of the identified biotic stress-responsive *V. vinifera* lncRNAs, pathways enrichment analysis was conducted. In response to both the biotrophs, potential involvement of lncRNAs was observed in plant hormone signal transduction pathways. Particularly, PM-responsive lncRNAs were found to be associated with salicylic acid (SA)-mediated *PR-1* induction, which is capable of enhancing resistance. Moreover, SA-signaling loop has been associated with cell death and hence, is involved in response to biotrophs [44]. Nevertheless, lncRNAs in response to the oomycete biotroph, that is, *P. viticola*, were observed to be associated with Jasmonate ZIM-domain (*JAZ*) that acts as a repressor for jasmonic acid (JA)-mediated signaling. JA has been reported to be related with resistance against *P. viticola* in grapevine [45]. Additionally, DM-responsive lncRNAs were found to be associated with brassinosteroid-promoted xyloglucan endotransglycosylases (*XET*) expressed by *TCH* (for touch) genes [46, 47]. As xyloglucan is the main hemicellulose in grapevine cell wall [48], their modulation by cell-wall modifying enzymes like XETs and co-expressing lncRNAs could possibly regulate cell wall reinforcement [49]. Overall, the results indicate an intricately regulated basal defense response mediated by the plausible association of PM- and DM-responsive lncRNAs with phytohormonal-based signal transduction in *V. vinifera*. Likewise, co-expressing lncRNA-CDS pairs were observed to be potentially involved in secondary metabolism pathways of grapevine, which further highlight the underlying regulation of plant response to the biotrophic phytopathogens.

As regulatory molecules, lncRNAs coordinate with other regulators of gene expression such as TFs and small ncRNAs such as miRNAs [24]. Also, as discussed earlier, in response to PM and DM, *V. vinifera* undergoes considerable transcriptional reprogramming; however, the underlying mechanisms remain obscure. Therefore, we revisited the interaction of regulatory players in view of lncRNAs. In response to both the biotrophs, lncRNAs were found to co-express with *WRKY* TFs, which have been reported to act as negative/positive regulators of plant defense including both the basal (pathogen-associated molecular patters [PAMP]-triggered immunity, PTI) and specific (effector-triggered immunity, ETI) immunity [50]. For instance, in response to PM, co-expressing ‘lncRNA and *probable WRKY70*’ pair was observed. Subject to induction by SA, *WRKY70* has been associated with *PR1* expression in response to an obligate biotrophic infection [44, 51]. Moreover, *WRKY70* has been associated with orchestrating cross-talks between phytohormones: SA and JA, which can act both antagonistically and synergistically to modulate local and systemic defense responses [32, 52]. This illustrates the potential of lncRNAs as important regulatory nodes in plant response to biotrophs.

In addition to the TF-mediated transcriptional-level regulation, lncRNAs can potentially coordinate regulation at post-transcriptional levels via their interactions with miRNAs. Generally, TF mRNAs are deemed as most common targets of miRNAs [53]. Interestingly, lncRNAs have been reported to be involved in target mimicry that facilitates inhibition of miRNA activity owing to its sequestration [54]. In the present study, we found some lncRNAs as putative eTMs for miRNAs whose original targets are TFs mRNAs. For instance, *V. vinifera* lncRNAs in response to both PM and DM were observed to act as putative eTMs for vvi-miR159c, which has been known to target *MYB TFs* [15]*.* Members of this TF family regulate flavonoid biosynthesis in grapevine [55] and have been associated with basal immunity in response to DM [56]. Overall, the interplay of lncRNAs, miRNAs, and TFs reflects the underlying sophistication involved in regulation of plant response to biotrophic pathogens like PM and DM. Furthermore, we found putative eTMs for vvi-miR482. This miRNA superfamily has been reported to target disease resistance-associated *Nucleotide Binding Site-Leucine Rich Repeats* (*NBS-LRR*) mRNAs in plants such as *S. lycopersicum* (tomato) and *Gossypium* sp. (cotton); however, upon fungal, bacterial or viral pathogenic attack, a suppression of the miRNA-driven silencing cascade has been reported [57, 58]. Therefore, further experiments could be conducted to explore the role of the identified putative eTMs in suppressing the aforementioned cascade via target mimicry to effectively induce the expression of *NBS-LRR* genes in *V. vinifera* in response to pathogens.

Lastly, qRT-PCR based analysis of high-confidence *V. vinifera* lncRNA candidates showed their differential response to DM at early (1 dpi) and advanced (3 dpi) stages compared to control. Interestingly, these lncRNAs were associated with CDS corresponding to defense response, for example, endo-1,3-beta-glucosidase and Barwin domain containing PR-4. Moreover, qRT-PCR based analysis confirmed similar co-expression trends with the corresponding defense-responsive protein CDS like PR-4, endochitinase precursor, and strigolactone esterase. Taken together, the present study forms a comprehensive repertoire of plausible lncRNA-mediated regulatory roles in response to biotrophic fungal and oomycete pathogens in *V. vinifera*. In future, similar studies can be conducted with an increased sample size of RNA-seq data to investigate the susceptible and resistant plants’ response against biotrophic pathogens at even later stages of infection; this would further enhance reliability and provide deeper insights. Based on this study, subsequent experiments can be conducted to explore lncRNAs as potent new candidates for engineering enhanced basal-resistance in the domesticated grapevine. Owing to the overlap in plant defense responses triggered by PTI and ETI against biotrophs, the understanding of lncRNA-mediated regulation in innate immunity can be extended to studying more specific defense responses as well.

## Methods

### Transcriptomic data collection

In order to investigate biotrophic stress-responsive lncRNAs in *V. vinifera*, transcriptomic data were collected corresponding to leaf samples infected by fungus- *E. necator* (powdery mildew; PM) and oomycete- *P. viticola* (downy mildew; DM) based on earlier studies using National Center for Biotechnology Information (NCBI) Sequence Read Archive (SRA) (http://www.ncbi.nlm.nih.gov/sra) [59, 60]. The details of the collected RNA-seq data have been provided in Additional File 1: Table S1.

### Differential expression analysis of transcripts

To understand the response of lncRNAs to PM and DM in *V. vinifera*, we performed the differential expression analysis of our previously identified 56,441 lncRNAs [16] using the aforementioned collected RNA-seq data. Expression levels of 37,420 V*. vinifera* coding sequences (CDS) were also determined. Firstly, RNA-Seq by Expectation–Maximization (RSEM) software (included within Trinity package v2.4.0) was used for transcript quantification, that is, the numbers of RNA-seq fragments per kilobase of transcript effective length per million fragments mapped to all transcripts (FPKM) were calculated. Next, the differentially expressed (DE) transcripts across the samples were analyzed using Empirical analysis of Digital Gene Expression data in R (edgeR). The DE transcripts that exhibited at least fourfold change at *P*-value cut-off (for false discovery rate [FDR]) <  = 0.01 with respect to their corresponding controls were selected. The distribution of statistically significant lncRNAs in response to PM and DM were visualized using volcano plots. Finally, the PM- and DM- responsive lncRNAs and CDS were analyzed for their expression patterns using heat maps generated by Hierarchical Clustering Explorer v3.5 (http://www.cs.umd.edu/hcil/hce).

### Functional annotation of the differentially expressed lncRNAs

The identified PM- and DM- responsive lncRNAs were functionally annotated based on co-expression analysis with respect to differentially expressed CDS (observed in the respective infections). The initial step was conducted using a bioinformatics tool- CoExpress v1.5 [61] and an in-house PERL script (https://github.com/ShivalikaP/Perl-script-tocalculate-Pearson-correlation-coefficient) [62] to calculate Pearson-correlation coefficient based on the expression data (FPKM) and identify the positively and negatively correlated co-expressing lncRNA and CDS pairs. The networks representing potential interactions between lncRNAs and CDS pairs based on co-expression were constructed using the strategy described by Pathania and Acharya, 2016 [62]. The next step included Blast2GO software (now a part of OmicsBox) [20] for gene ontology (GO) enrichment analysis of CDS co-expressing with the respective PM- and DM- responsive lncRNAs (at Pearson-correlation coefficient threshold of 0.9). Next, pathways enrichment analysis was conducted for the co-expressing CDS with the aid of a web server, KEGG Orthology Based Annotation System or KOBAS 3.0 [63] using Kyoto Encyclopaedia of Genes and Genomes (KEGG) pathways database [21] exclusively for the species: *Vitis vinifera*.

### Identification of transcription factors co-expressing with lncRNAs in response to PM and DM

The PM- and DM-responsive CDS, which were found to co-express with lncRNAs (as described above) were screened for transcription factors (TFs). For this, TF prediction server based on Plant TF database v5.0 (PlantTFDB) [64] was used. This tool is dependent on ESTScan 3.0 [65] for Hidden Markov Model (HMM)-based analysis of coding regions in the provided input sequences.

### Interaction analysis of lncRNAs and miRNAs

To conduct the interaction analysis of the identified PM- and DM-stress responsive lncRNAs with miRNAs, the latter were obtained from miRNA database (miRBase) [66] specifically for *V. vinifera*. Firstly, a bioinformatics tool, plant small RNA target analysis server (psRNATarget) was used with default parameters to identify target sites of *V. vinifera* mature miRNAs in the identified lncRNAs. Next, another tool, TAPIR (http://bioinformatics.psb.ugent.be/webtools/tapir/) [67] was used to predict endogenous target mimics (eTMs) at an mfe_ratio >  = 0.5 for both PM- and DM-responsive lncRNAs. Further, minimum free energy secondary structures for lncRNAs were analyzed and visualized using Vienna RNAfold web server (http://rna.tbi.univie.ac.at/) [68] and forna tool [69]. Finally, Gephi (https://gephi.org/) [70] was used to gain an overview of the interactions between lncRNAs and miRNAs.

### qRT-PCR-based expression analysis of lncRNAs

To validate the expression of lncRNAs in response to biotic stress, leaf samples of *V. vinifera* cv. Thompson seedless were collected from the vineyards at Indian Council of Agricultural Research-National Research Centre for Grapes (ICAR-NRCG), Pune, India. Leaf samples corresponding to control, early (1 dpi) and late (3 dpi) infection stages of DM were harvested using liquid nitrogen. Total RNA was extracted for the samples using a protocol standardized for plant tissues rich in secondary metabolites [71]. The extracted RNA was treated with DNase I (Amplification grade, Invitrogen, USA) for the removal of contaminating genomic DNA. Next, cDNA was prepared using Superscript III first strand cDNA synthesis kit (Invitrogen USA). The primers for qRT-PCR analysis were designed using Primer3 Input software [72] (Additional File 2: Table S2) and the subsequent PCR were performed using Bio-Rad CFX96™ Real-Time PCR system. For normalization of gene expression, *Actin7* (*ACT7*) (NCBI reference sequence ID: XM_002282480.4) was used as an internal control gene. For calculating the relative gene expression, 2^–ΔΔCT^ method was applied [73]. All experiments were conducted in triplicates. Statistical analysis was performed based on unpaired *t-*test by using GraphPad Prism software (GraphPad Software, Inc. La Jolla, CA).

## Conclusions

In order to understand the underlying regulation of plant response to obligate biotrophic fungal phytopathogens, we conducted genome-wide analysis using computational approach to identify 71 and 83 *Vitis vinifera* (grapevine) lncRNAs in response to *Erysiphe necator* (powdery mildew, PM) and *Plasmopara viticola* (downy mildew, DM), respectively. Expression profiling for *V. vinifera* protein coding sequences (CDS) was also conducted, and 1037 PM-responsive and 670 DM-responsive CDS were identified. A comprehensive functional annotation analysis was conducted for the identified lncRNAs based on their co-expression with these responsive CDS. The analysis revealed their association with Ca^2+^-binding proteins such as calmodulin/calmodulin-like proteins, enzymes involved in reactive oxygen species (ROS) metabolism, cell-wall modification/reinforcement, secondary metabolic pathways, phytoalexin (like resveratrol) production, pathogenesis-related proteins such as PR-1, PR-4 and PR-10, and phytohormone-based signal transduction. Moreover, lncRNA-miRNA interaction network analysis revealed the possibility of target mimicry in regulation of the underlying mechanisms of plant defense response. Transcription factors (TFs) such as WRKY, which regulate both basal (PTI) and pathogen-specific defense responses (ETI) were also found to be associated with candidate lncRNAs in response to both PM and DM. Overall, as regulatory molecules, PM- and DM- responsive lncRNAs can coordinate with other regulators of gene expression and facilitate transcriptional reprogramming in response to the biotrophic pathogens in *V. vinifera*. In view of their regulatory versatility, the identified lncRNAs such as those for which quantitative polymerase chain reaction analysis was conducted in this study or which were identified as potent nodes in miRNA-mediated cascade regulation can be further examined. Such lncRNAs upon subsequent investigation can be leveraged as candidates for biotechnological improvement of the susceptible grapevine crop in addition to the conventional approach of over-expressing defense-related genes or TFs.

## Supplementary Information

**Additional file 1: Table S1.** Details of transcriptomic data collected from NCBI-SRA database.

**Additional file 2: Table S2.** List of Primers used for qRT-PCR. **Figure S1.** Expression profiles of lncRNAs in response to (A) *Erysiphe necator* (powdery mildew) infection at 36 hpi (4-fold change, *P*-value <= 0.01) and (B) *Plasmopara viticola* (downy mildew) infection at 24 and 48 hpi (4-fold change, *P*-value<= 0.01). The bigger clusters of lncRNAs based on expression trends have been shown in red font color, while those in blue represent the smaller groups. More up-regulated and down-regulated lncRNAs are observed in response to (A) PM infection and (B) DM infection, respectively. The color scale corresponds to log ratio of expression (FPKM). A high value has a bright red color and a low value has bright green color. The middle value has a black color. hpi, hours post inoculation; PM, powdery mildew; DM, downy mildew. **Figure S2.** Expression Profile of Coding Sequences of *Vitis vinifera* in response to (A) *Erysiphe necator* (powdery mildew, PM) infection at 36 hpi and (B) *Plasmopara viticola* (downy mildew, DM) infection at 24 and 48 hpi. The color scale corresponds to log ratio of expression (FPKM). A high value has a bright red color and a low value has bright green color. The middle value has a black color. hpi, hours post inoculation **Figure S3.** (A) PM- and DM-responsive lncRNAs have only one transcript in common. (B) DM- and PM-responsive CDS have 94 transcripts in common. **Figure S4.** Topological analysis of lncRNAs-CDS co-expression network to determine the Pearson correlation coefficient (PCC) threshold based on Network density (ND) in (A) Powdery Mildew and (B) Downy Mildew. Where, PCC corresponding to this minimal ND is depicted in diamond shape and considered as the threshold (0.90). The in-house script that was used for this analysis can be found at: GitHub (https://github.com/ShivalikaP/Perl-script-tocalculate-Pearson-correlation-coefficient). **Figure S5.** Co-expression based network of DM-responsive lncRNAs and CDS: The Co-expression network comprising lncRNAs and associated CDS with red and blue interactions represents the positive and negative correlations, respectively. In addition, the nodes in green and purple colors with diamond and circle shapes are representing lncRNAs and the associated CDS, respectively. **Figure S6.** Complete weighted DM-responsive lncRNAs-CDS network, which is obtained from integration of weighted CDS-CDS and lncRNAs-CDS (with positive correlations) co-expression network. The lncRNAs and CDS are depicted as diamond and circle shapes in purple and pink colors (with edges as solid lines), respectively. **Figure S7.** Complete weighted DM-responsive lncRNAs-CDS network, which is obtained from integration of weighted CDS-CDS and lncRNAs-CDS (with negative correlations) co-expression network. The lncRNAs and CDS are depicted as diamond and circle shapes in purple and pink colors (with edges as solid lines), respectively. **Figure S8.** InterProScan (IPS) sites distribution for coding sequences coexpressing with (A) DM- and (B) PM-responsive lncRNAs. **Figure S9.** Number of pathways observed during enrichment analysis for mRNAs co-expressing with lncRNAs in response to different PM, powdery mildew and DM, downy mildew. **Figure S10.** Potential involvement of lncRNAs (co-expressing with mRNAs) in ‘plant-pathogen interaction’ pathway in (A) powdery mildew and (B) downy mildew infections highlighted in red color. **Figure S11.***V. vinifera* miRNAs potentially targeting the identified PM- and DM-responsive lncRNAs. **Figure S12.***V. vinifera* miRNAs for which the identified PM- and DM-responsive lncRNAs can act as potential endogenous target mimics (eTMs). **Figure S13.** Comparative analyses of RNA-seq and qRT-PCR data for the selected high-confidence lncRNAs at the common time point of DM infection, that is, 24 hpi or 1 dpi. Expression levels have been represented as log natural fold change values. **Figure S14.** Co-expression patterns of selected DM-responsive lncRNAs and corresponding protein coding sequences (CDS). (A-D) depict the co-expression patterns of 4 DM-responsive lncRNAs- CDS pairs. The blue and red colors correspond to expression patterns observed by *in silico* differential expression analysis (FPKM values); while green and purple represent expression trends observed after qRT-PCR analysis. The names of the lncRNAs and NCBI reference sequence IDs of the CDS are provided in the color legends in each panel. The y-axis corresponds to the natural logarithm of the fold change values.

**Additional file 3: **Co-expressing powdery and downy mildew-responsive *Vitis vinifera* lncRNAs and mature mRNAs at Pearson correlation coefficient >= 0.9 (using CoExpress v1.5), including all the positively and negatively co-related co-expressing pairs based on Pearson correlation coefficient using the PERL Script.

**Additional file 4:** Gene Ontology distribution for all 3 categories for mRNAs coexpressing with PM and DM-responsive lncRNAs and direct Gene Ontology (GO) Count representing the most frequent GO terms in the Biological Processes category.

**Additional file 5:** Pathway enrichment analysis for lncRNAs co-expressing with CDS in response to powdery and downy mildew.

**Additional file 6: **PM- and DM-responsive lncRNAs as putative targets and endogenous target mimics of *Vitis vinifera* miRNAs. PM- and DM-responsive protein coding sequences (which are coexpressing with PM- and DM-responsive lncRNAs) as putative targets of *Vitis vinifera* miRNAs.

**Additional file 7: **The interaction analyses of the PM- and DM-responsive lncRNAs with *V. vinifera* miRNAs to gain an overview of the interactome.

## Data Availability

The datasets analyzed during the current study are available in the NCBI SRA repository, (http://www.ncbi.nlm.nih.gov/sra). The details have been included in Additional File 1: Table S1. All the 56,441 *Vitis vinifera* lncRNAs have been provided in this published article: Bhatia, *et al.* 2019 (https://doi.org/10.1038/s41598-019-38989-7) and its supplementary data file 2.

## Abbreviations

BLAST: Basic local alignment search tool
BP: Biological processes
CAP: Cysteine-rich secretory proteins
CC: Cellular component
CDS: Coding sequences
CWDE: Cell-wall degrading enzymes
DM: Downy mildew
dpi: Days post inoculation
EC: Enzyme codes
ETI: Effector-triggered immunity
eTMs: Endogenous target mimics
FDR: False discovery rate
GO: Gene ontology
HMM: Hidden Markov Model
hpi: Hours post inoculation
JA: Jasmonic acid
JAZ: Jasmonate ZIM-domain
KEGG: Kyoto Encyclopedia of Genes and Genomes
KOBAS: KEGG Orthology Based Annotation System
lncRNAs: Long non-coding RNAs
MF: Molecular functions
miRNAs: Micro RNAs
NBS-LRR: Nucleotide Binding Site-Leucine Rich Repeats
nt: Nucleotides
PM: Powdery mildew
PR: Pathogenesis-related proteins
psRNATarget: Plant small RNA target analysis server
PTI: Pathogen-associated molecular patters (PAMP)-triggered immunity
qRT-PCR: Quantitative real time polymerase reaction
ROS: Reactive oxygen species
SA: Salicylic acid
TAIR: The Arabidopsis Information Resource
TFs: Transcription factors
XET: Xyloglucan endotransglycosylases
