# Supplementary material for: Investigation of long non-coding RNAs as regulatory players of grapevine response to powdery and downy mildew infection
Source: BMC Plant Biol. 2021 Jun 8;21:265. doi: 10.1186/s12870-021-03059-6 (PMC8186045; doi:10.1186/s12870-021-03059-6)
Supplement: Supplementary file 2 — Additional file 2: Table S2. List of Primers used for qRT-PCR. Figure S1. Expression profiles of lncRNAs in response to (A) Erysiphe necator (powdery mildew) infection at 36 hpi (4-fold change, P-value <= 0.01) and (B) Plasmopara viticola (downy mildew) infection at 24 and 48 hpi (4-fold change, P-value<= 0.01). The bigger clusters of lncRNAs based on expression trends have been shown in red font color, while those in blue represent the smaller groups. More up-regulated and down-regulated lncRNAs are observed in response to (A) PM infection and (B) DM infection, respectively. The color scale corresponds to log ratio of expression (FPKM). A high value has a bright red color and a low value has bright green color. The middle value has a black color. hpi, hours post inoculation; PM, powdery mildew; DM, downy mildew. Figure S2. Expression Profile of Coding Sequences of Vitis vinifera in response to (A) Erysiphe necator (powdery mildew, PM) infection at 36 hpi and (B) Plasmopara viticola (downy mildew, DM) infection at 24 and 48 hpi. The color scale corresponds to log ratio of expression (FPKM). A high value has a bright red color and a low value has bright green color. The middle value has a black color. hpi, hours post inoculation Figure S3. (A) PM- and DM-responsive lncRNAs have only one transcript in common. (B) DM- and PM-responsive CDS have 94 transcripts in common. Figure S4. Topological analysis of lncRNAs-CDS co-expression network to determine the Pearson correlation coefficient (PCC) threshold based on Network density (ND) in (A) Powdery Mildew and (B) Downy Mildew. Where, PCC corresponding to this minimal ND is depicted in diamond shape and considered as the threshold (0.90). The in-house script that was used for this analysis can be found at: GitHub (https://github.com/ShivalikaP/Perl-script-tocalculate-Pearson-correlation-coefficient). Figure S5. Co-expression based network of DM-responsive lncRNAs and CDS: The Co-expression network comprising lncRNAs and as [file 12870_2021_3059_MOESM2_ESM.docx]

| Table S2: List of Primers used for qRT-PCR | | |  |
| --- | --- | --- | --- |
|  |  | Fwd | Reverse |
| LncRNAs |  |  |  |
| TR39926 |  | GGTTGCTGCTCTCCATGTTT | ATAAAGTCAACCCGGCCATG |
| TR39929 |  | AGGGGTCTTCAATGGCTTCT | TTGTACTTGTAAACCGGCGG |
| TR41247 |  | CCTGTTCTAAATCCGCCTCA | CAGGAGGCTTCTTTTGTGGT |
| TR101084 |  | CACACCCTGATGCCTATTAAGT | AGTACTCCCAAGGATCACACC |
|  |  |  |  |
| Protein Coding Sequences |  |  |  |
| XP_002264720.1 |  | CGCAGCAAGTATGGATGGAC | GCCAGCCCCATTAGTGTCTA |
| NP_001268048.1 |  | CCCTAACCCAAACCTGCAAC | CTTCCAACCCCACCACCTAT |
| XP_010664515.1 |  | CGCTACACCACTCTTGATGC | AGGAATCTGGGAGAAGCACC |
|  |  |  |  |
| Internal control gene |  |  |  |
| XM_002282480.4 *Actin 7* | | GGTGATGATGCTCCCAGGGC | ACTGGGTGTTCTTCAGGGGC |


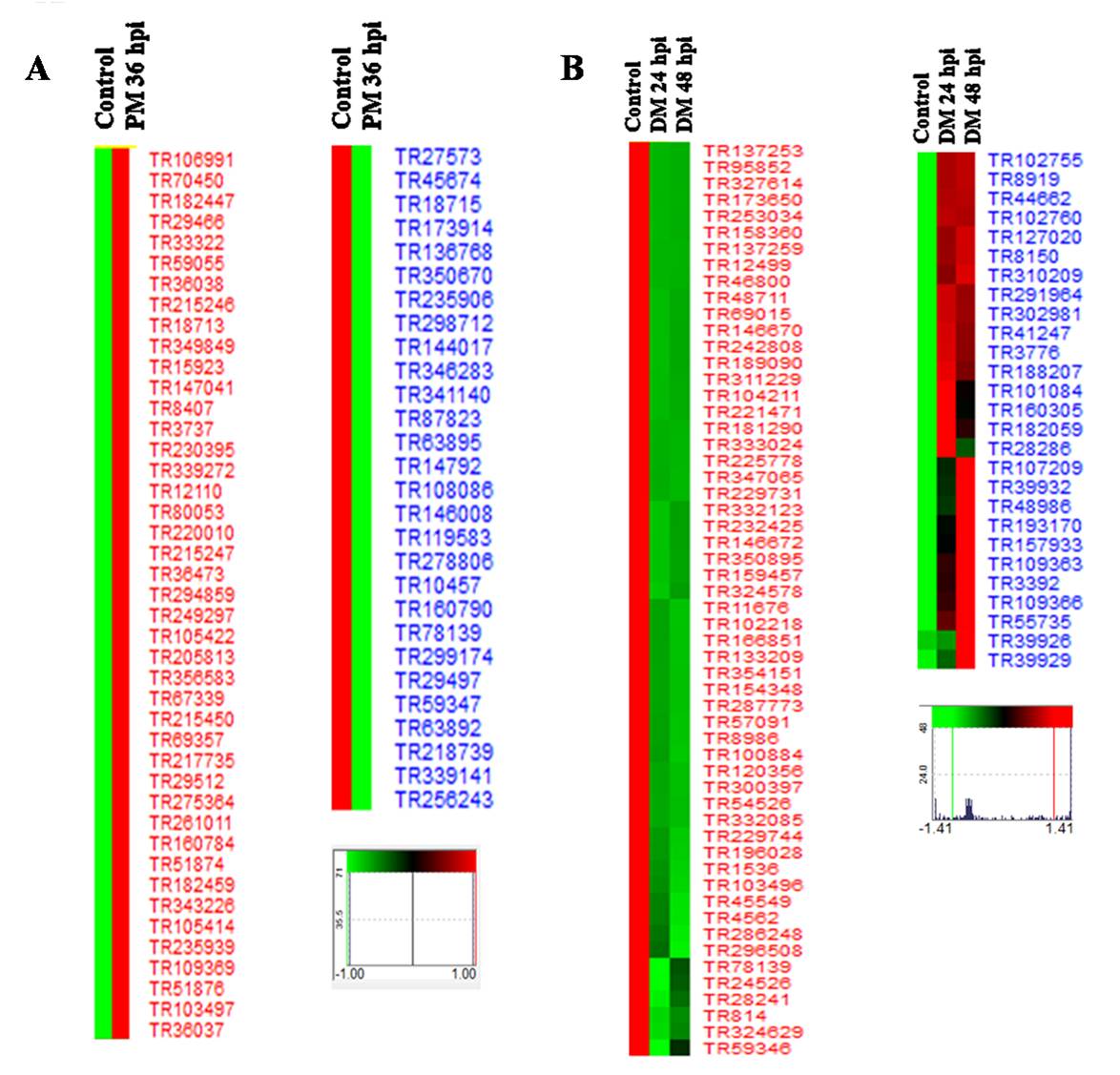


**Figure S1: Expression profiles of lncRNAs in response to (A) *Erysiphe necator* (powdery mildew) infection at 36 hpi (4-fold change, *P-*value<= 0.01) and (B) *Plasmopara viticola* (downy mildew) infection at 24 and 48 hpi (4-fold change, *P-*value<= 0.01).** The bigger clusters of lncRNAs based on expression trends have been shown in red font color, while those in blue represent the smaller groups. More up-regulated and down-regulated lncRNAs are observed in response to (A) PM infection and (B) DM infection, respectively. The color scale corresponds to log ratio of expression (FPKM). A high value has a bright red color and a low value has bright green color. The middle value has a black color. hpi, hours post inoculation; PM, powdery mildew; DM, downy mildew.


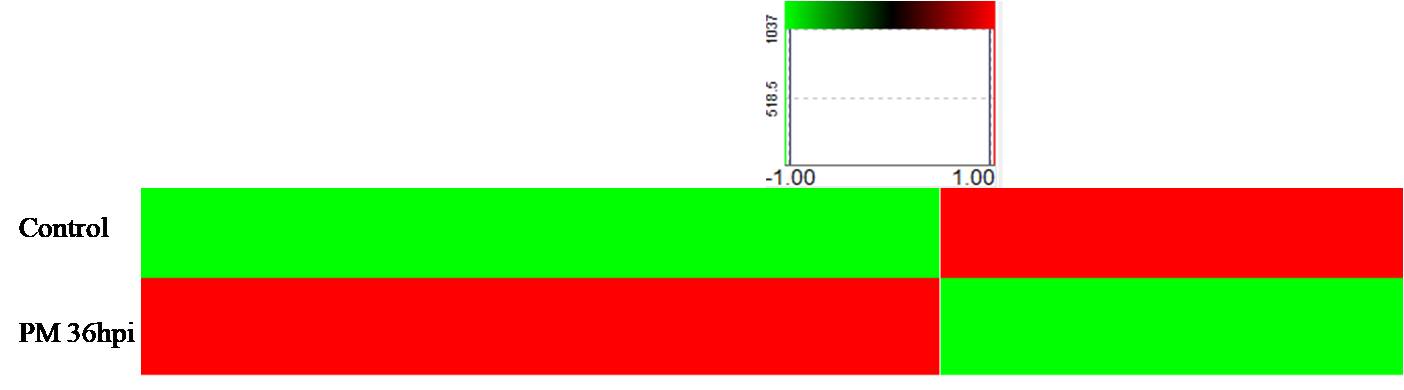


**A**


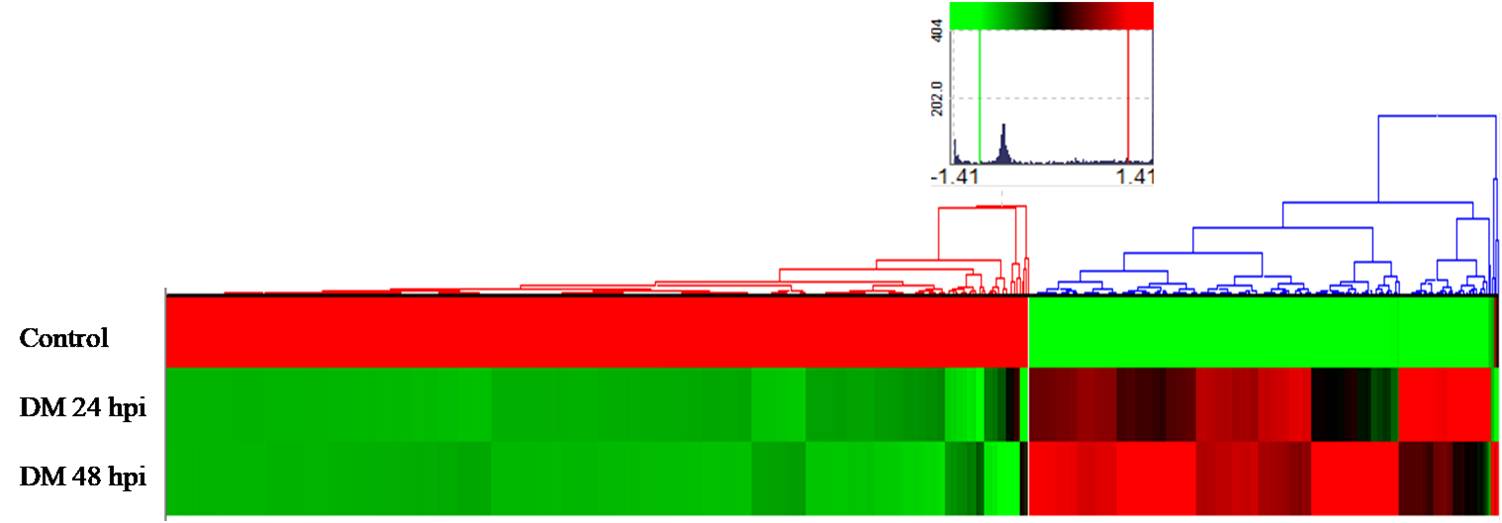


**B**

**Figure S2: Expression Profile of Coding Sequences of *Vitis vinifera* in response to (A) *Erysiphe necator* (powdery mildew, PM) infection at 36 hpi and (B) *Plasmopara viticola* (downy mildew, DM) infection at 24 and 48 hpi.** The color scale corresponds to log ratio of expression (FPKM). A high value has a bright red color and a low value has bright green color. The middle value has a black color. hpi, hours post inoculation

**
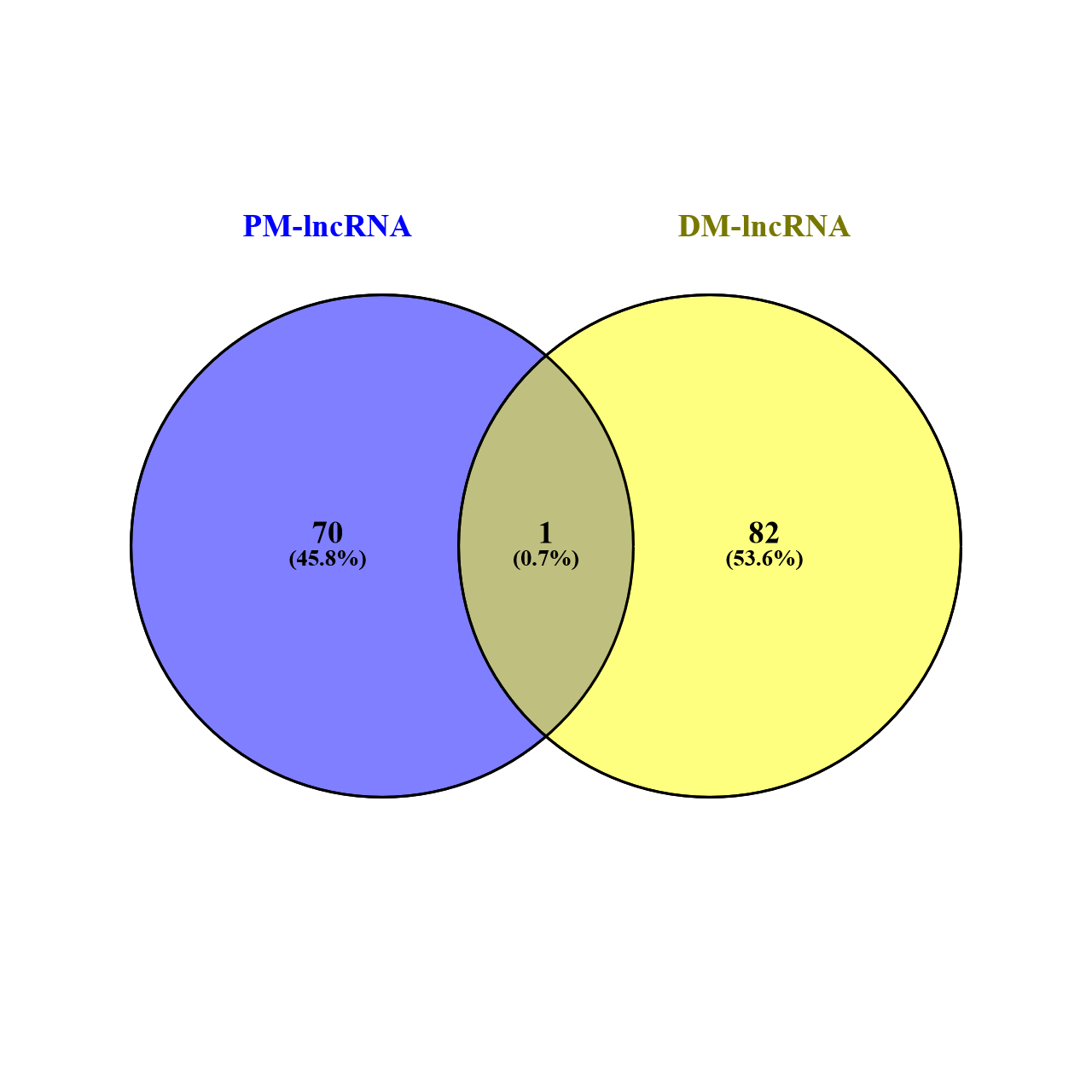
**

**A**

**
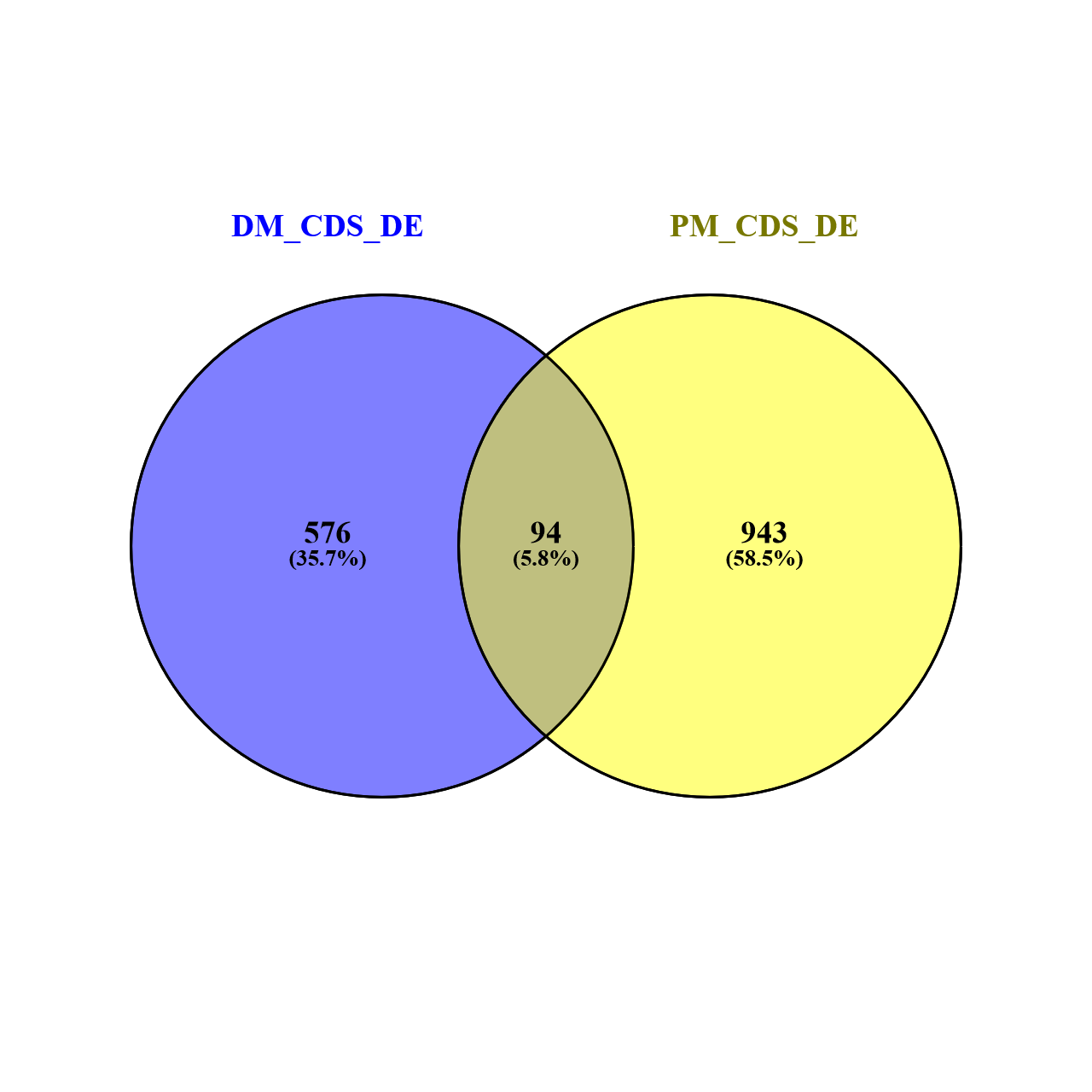
**

**B**

**Figure S3: (A) PM- and DM-responsive lncRNAs have only one transcript in common. (B) DM- and PM-responsive CDS have 94 transcripts in common.**


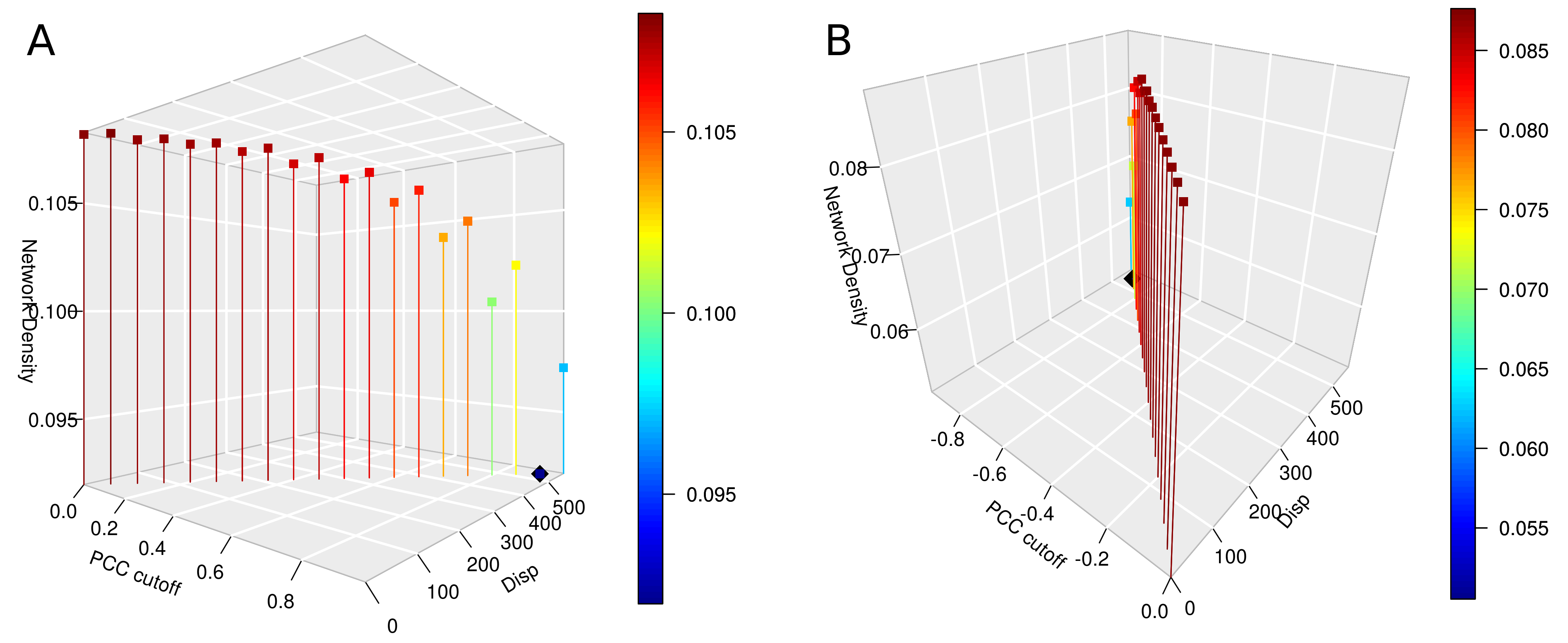


**Figure S4: Topological analysis of lncRNAs-CDS co-expression network to determine the Pearson correlation coefficient (PCC) threshold based on Network density (ND) in (A) Powdery Mildew and (B) Downy Mildew.** Where, PCC corresponding to this minimal ND is depicted in diamond shape and considered as the threshold (0.90). The in-house script that was used for this analysis can be found at: GitHub (https://github.com/ShivalikaP/Perl-script-tocalculate-Pearson-correlation-coefficient).

**
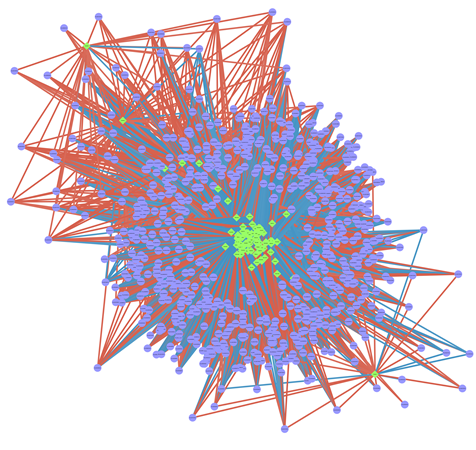
**

**Figure S5: Co-expression based network of DM-responsive lncRNAs and CDS:** The Co-expression network comprising lncRNAs and associated CDS with red and blue interactions represents the positive and negative correlations, respectively. In addition, the nodes in green and purple colors with diamond and circle shapes are representing lncRNAs and the associated CDS, respectively.


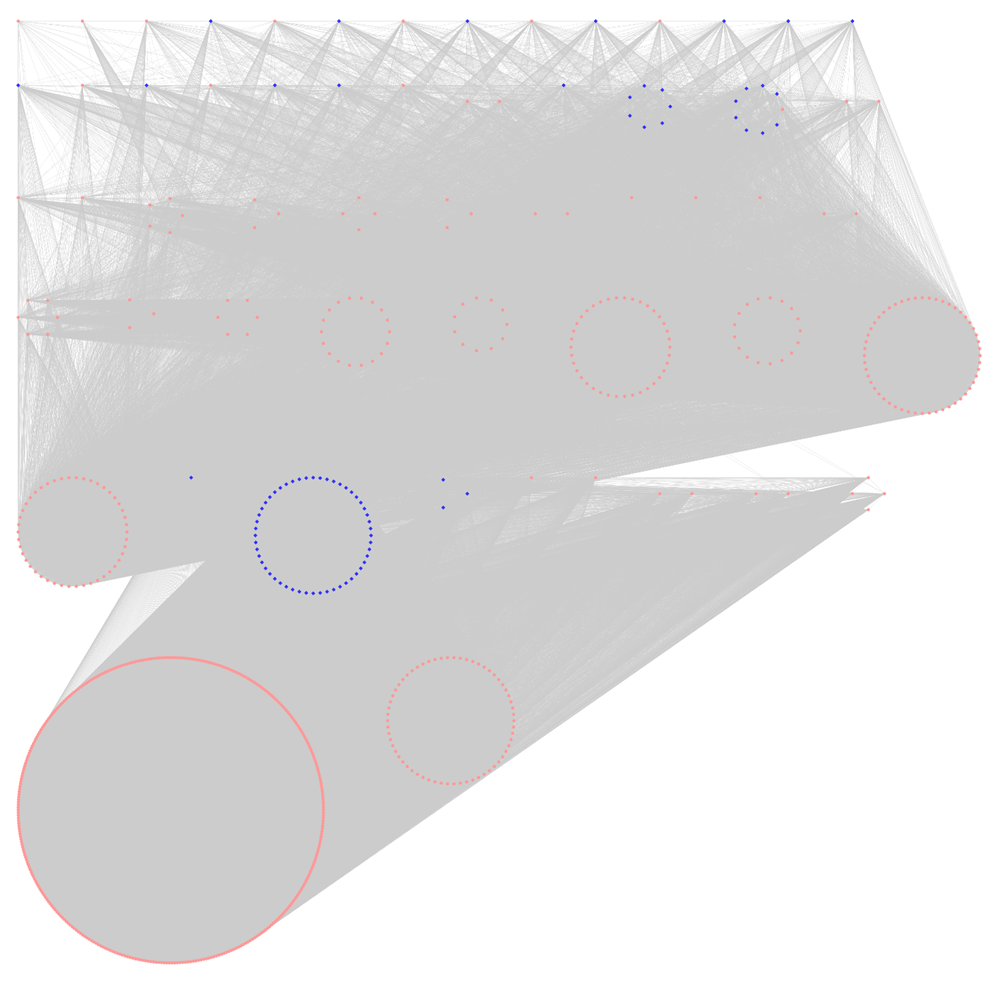


**Figure S6: Complete weighted DM-responsive lncRNAs-CDS network, which is obtained from integration of weighted CDS-CDS and lncRNAs-CDS (with positive correlations) co-expression network.** The lncRNAs and CDS are depicted as diamond and circle shapes in purple and pink colors (with edges as solid lines), respectively.

**
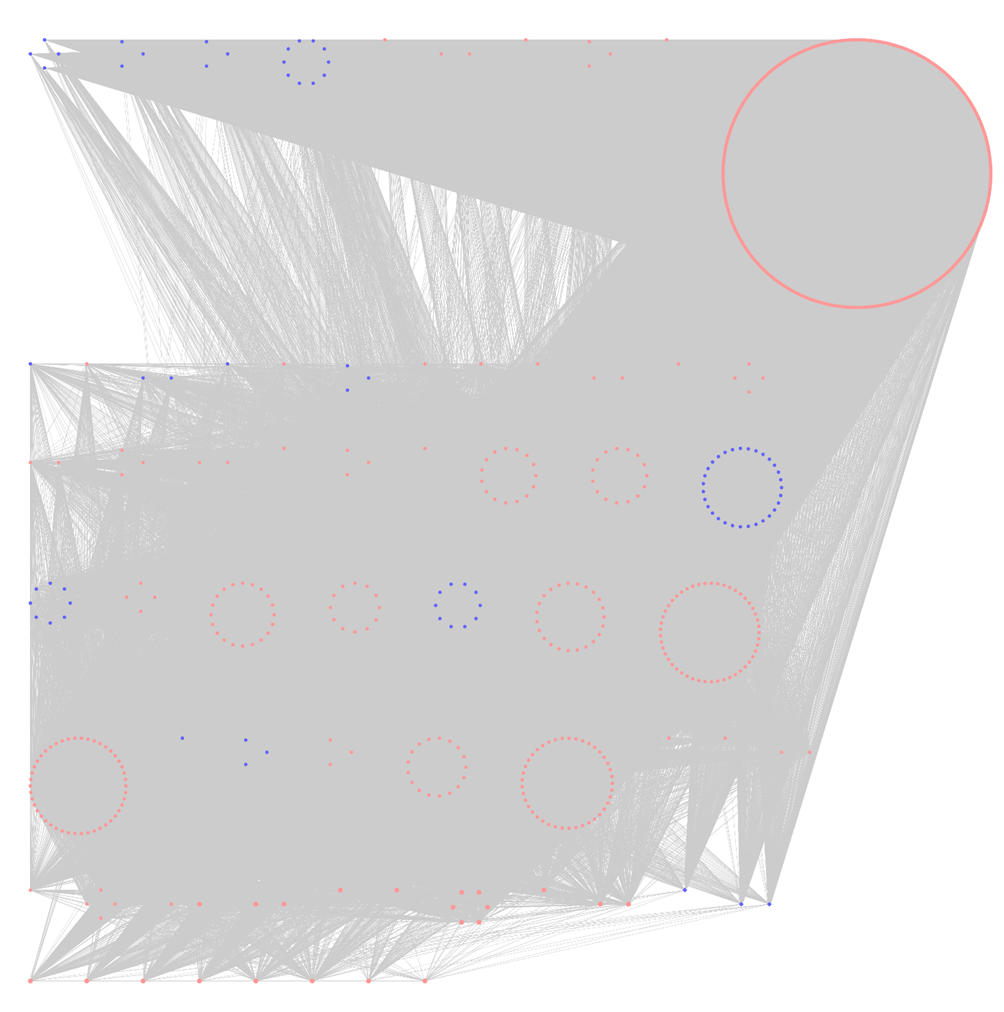
**

**Figure S7: Complete weighted DM-responsive lncRNAs-CDS network, which is obtained from integration of weighted CDS-CDS and lncRNAs-CDS (with negative correlations) co-expression network.** The lncRNAs and CDS are depicted as diamond and circle shapes in purple and pink colors (with edges as solid lines), respectively.


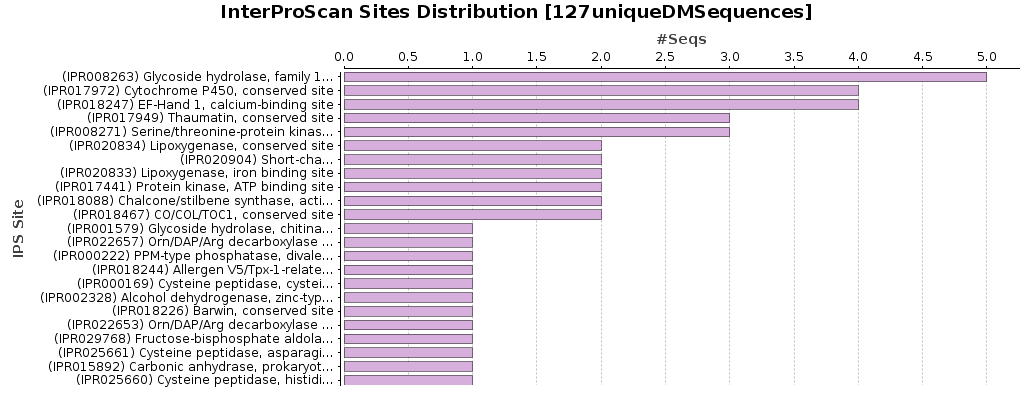


**A**

**B**


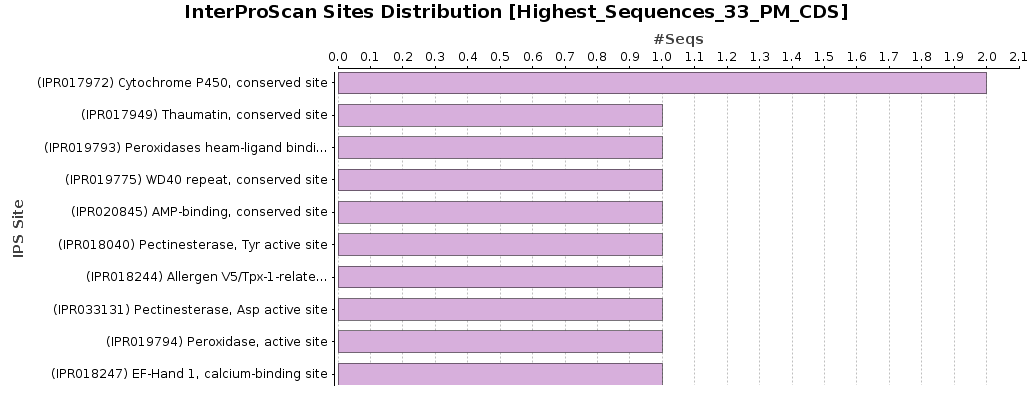


**Figure S8: InterProScan (IPS) sites distribution for coding sequences coexpressing with (A) DM- and (B) PM-responsive lncRNAs.**

**
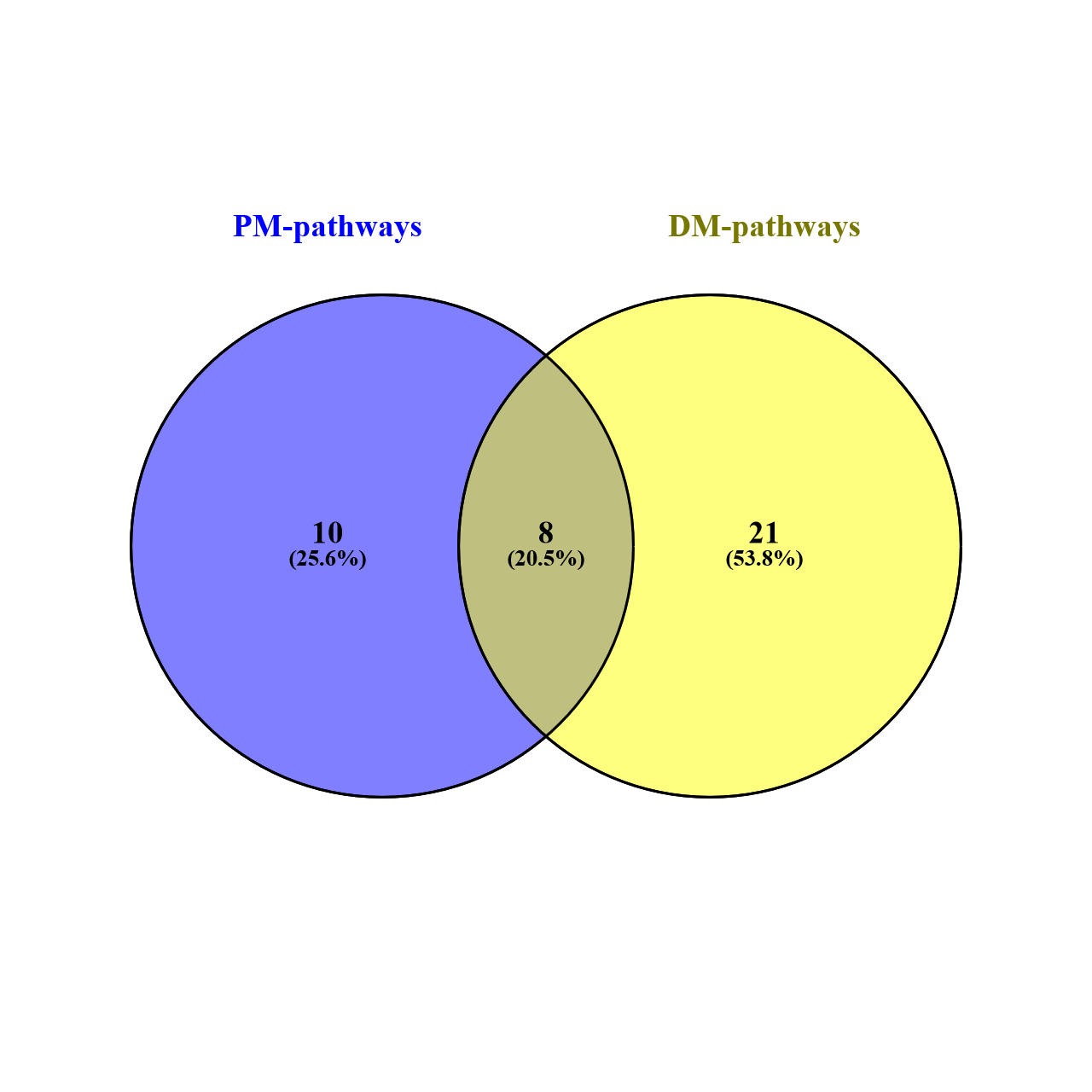
**

**Figure S9: Number of pathways observed during enrichment analysis for mRNAs coexpressing with lncRNAs in response to different PM, powdery mildew and DM, downy mildew.**

**
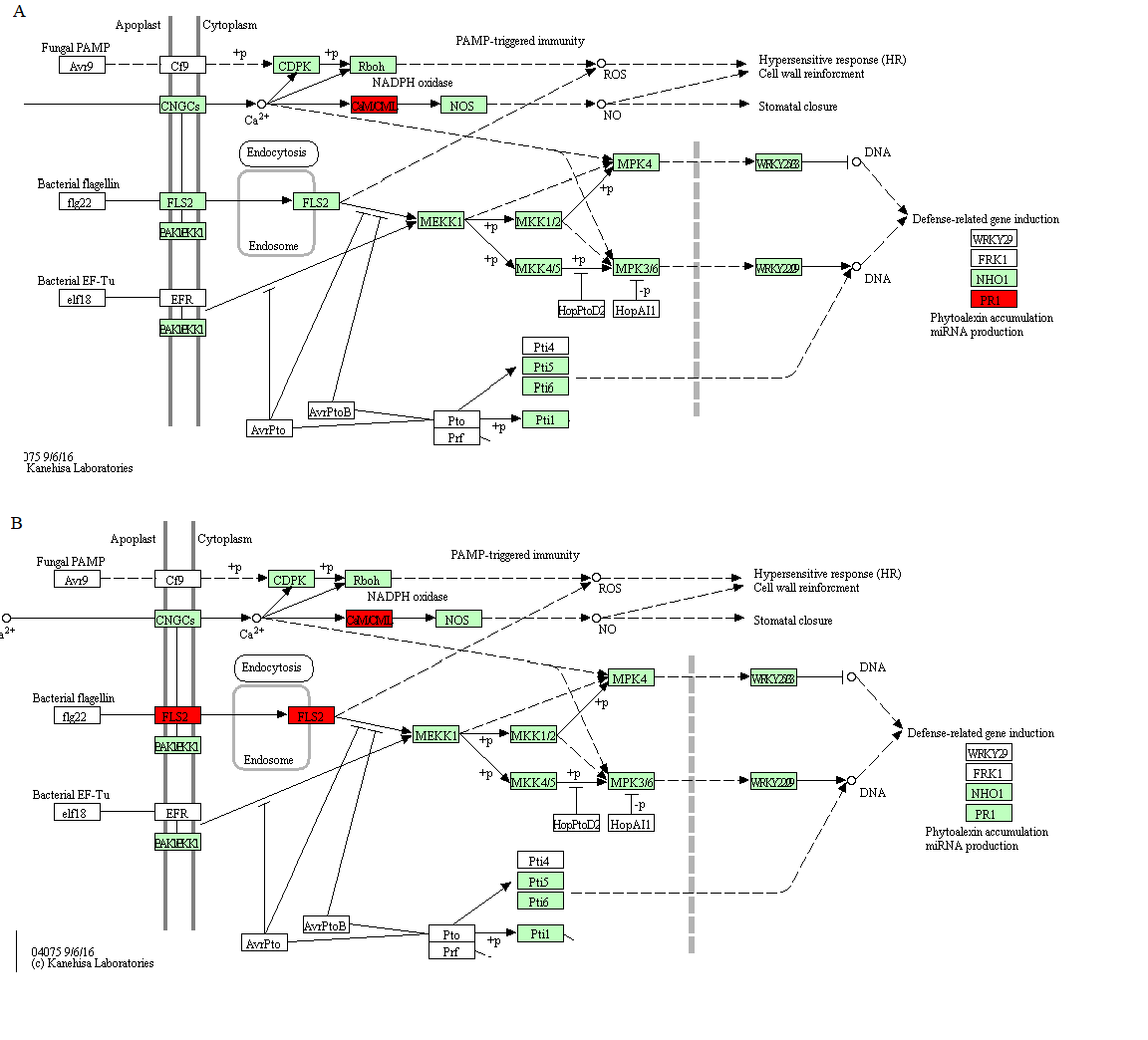
**

**Figure S10: Potential involvement of lncRNAs (co-expressing with mRNAs) in ‘plant-pathogen interaction’ pathway in (A) powdery mildew and (B) downy mildew infections highlighted in red color.**

**
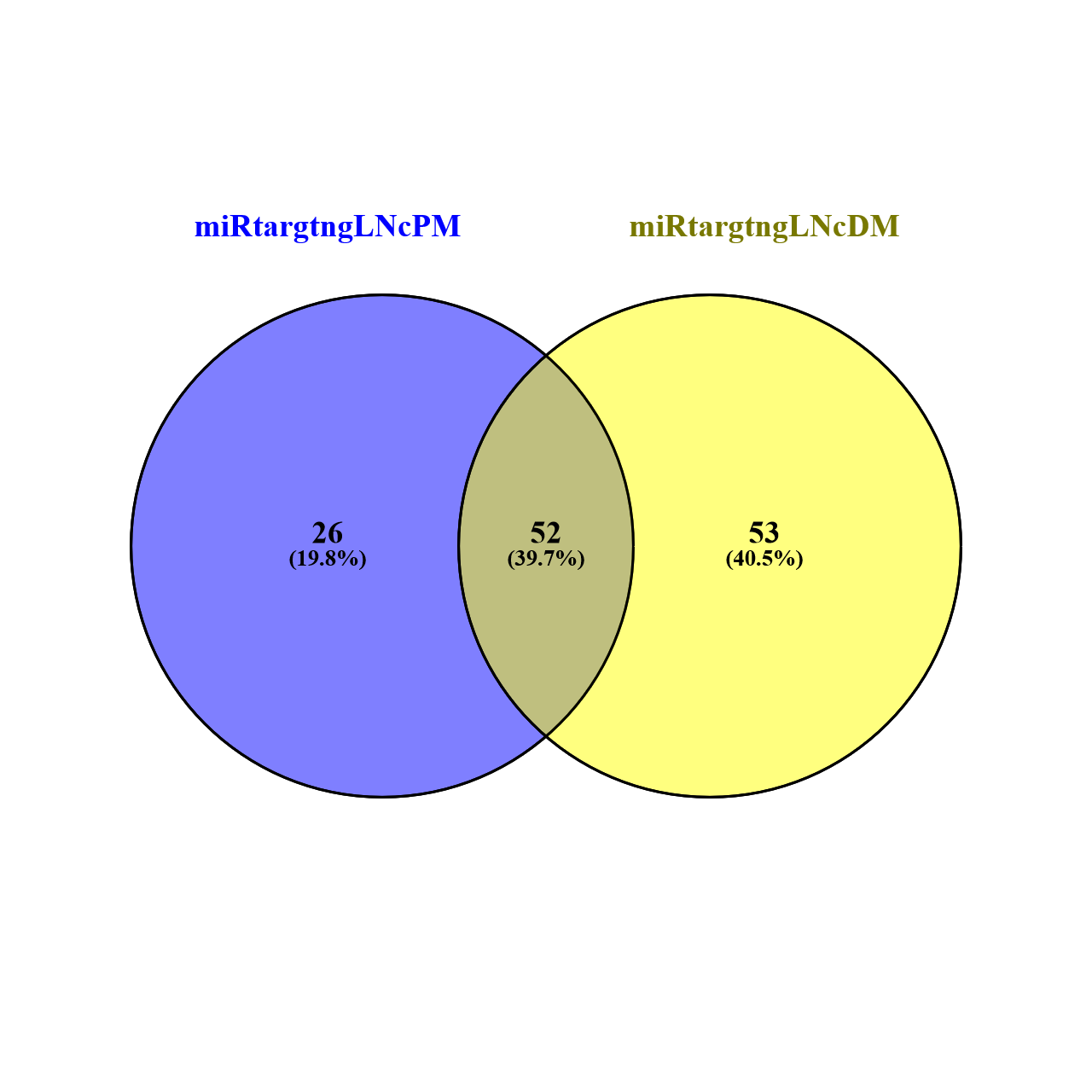
**

**miRNAs targeting DM-responsive lncRNAs**

**miRNAs targeting PM-responsive lncRNAs**

**Figure S11: *V. vinifera* miRNAs potentially targeting the identified PM- and DM-responsive lncRNAs**

**miRNAs for which PM-responsive lncRNAs act as eTMs**

**miRNAs for which DM-responsive lncRNAs act as eTMs**

**
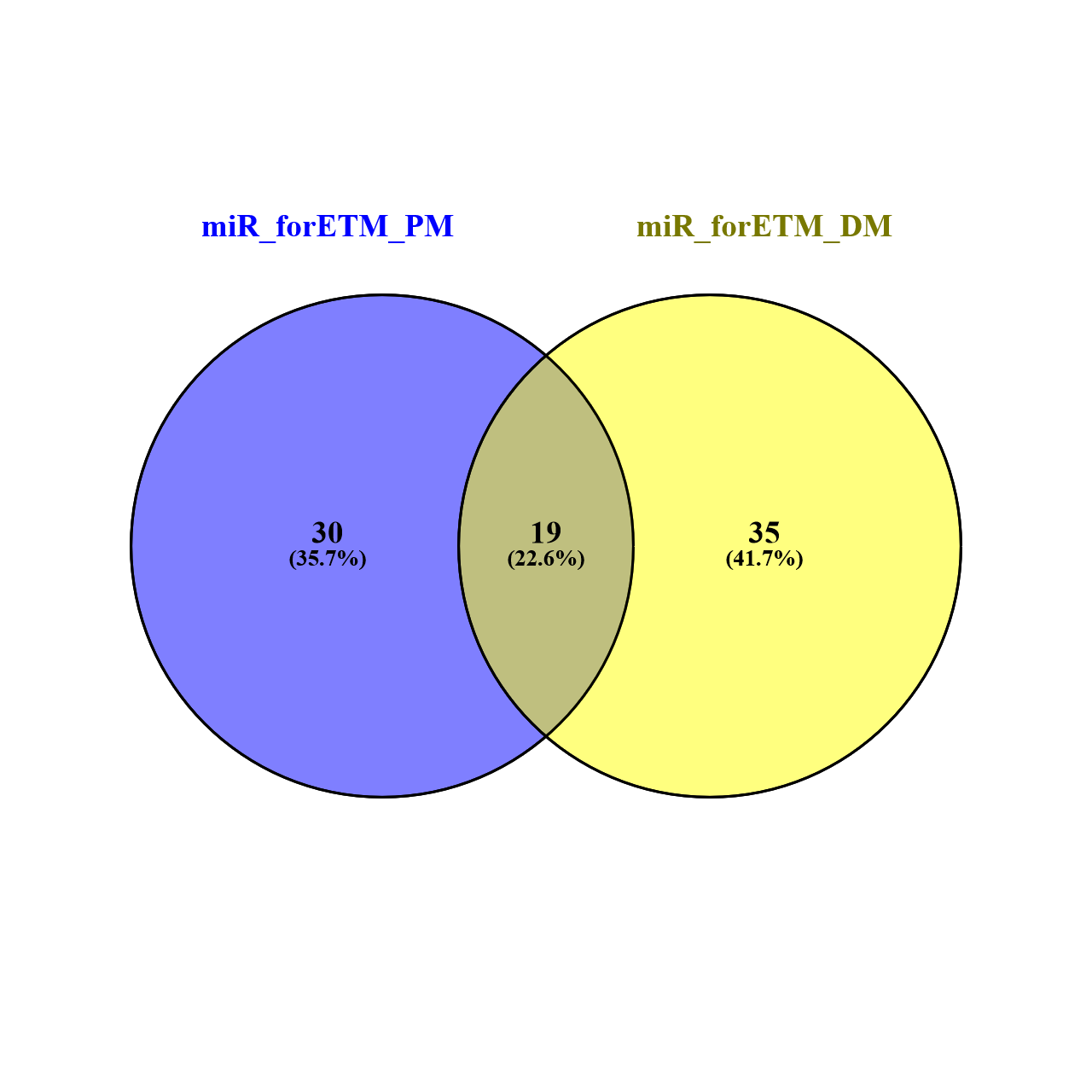
**

**Figure S12: *V. vinifera* miRNAs for which the identified PM- and DM-responsive lncRNAs can act as potential endogenous target mimics (eTMs)**


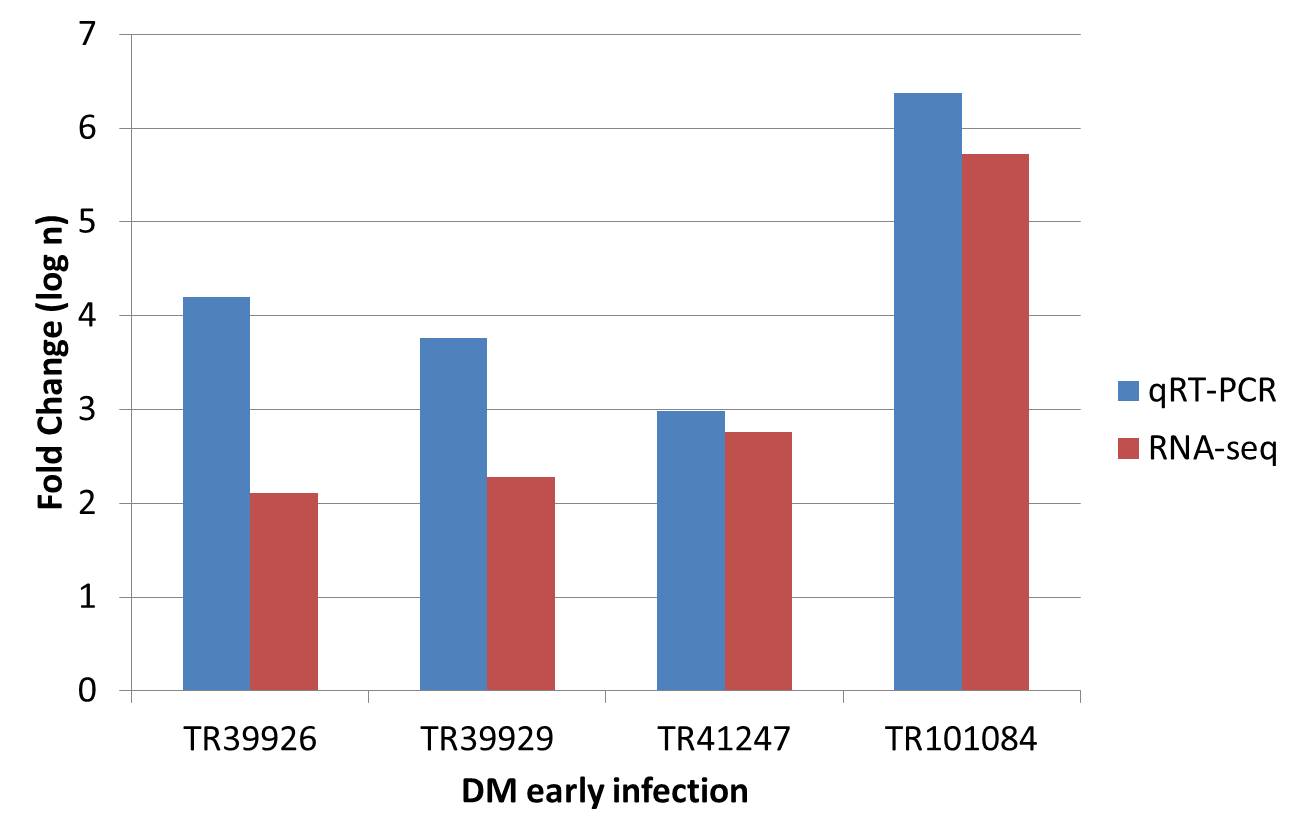


**Figure S13: Comparative analyses of RNA-seq and qRT-PCR data for the selected high-confidence lncRNAs at the common time point of DM infection, that is, 24 hpi or 1 dpi. Expression levels have been represented as log natural fold change values.**


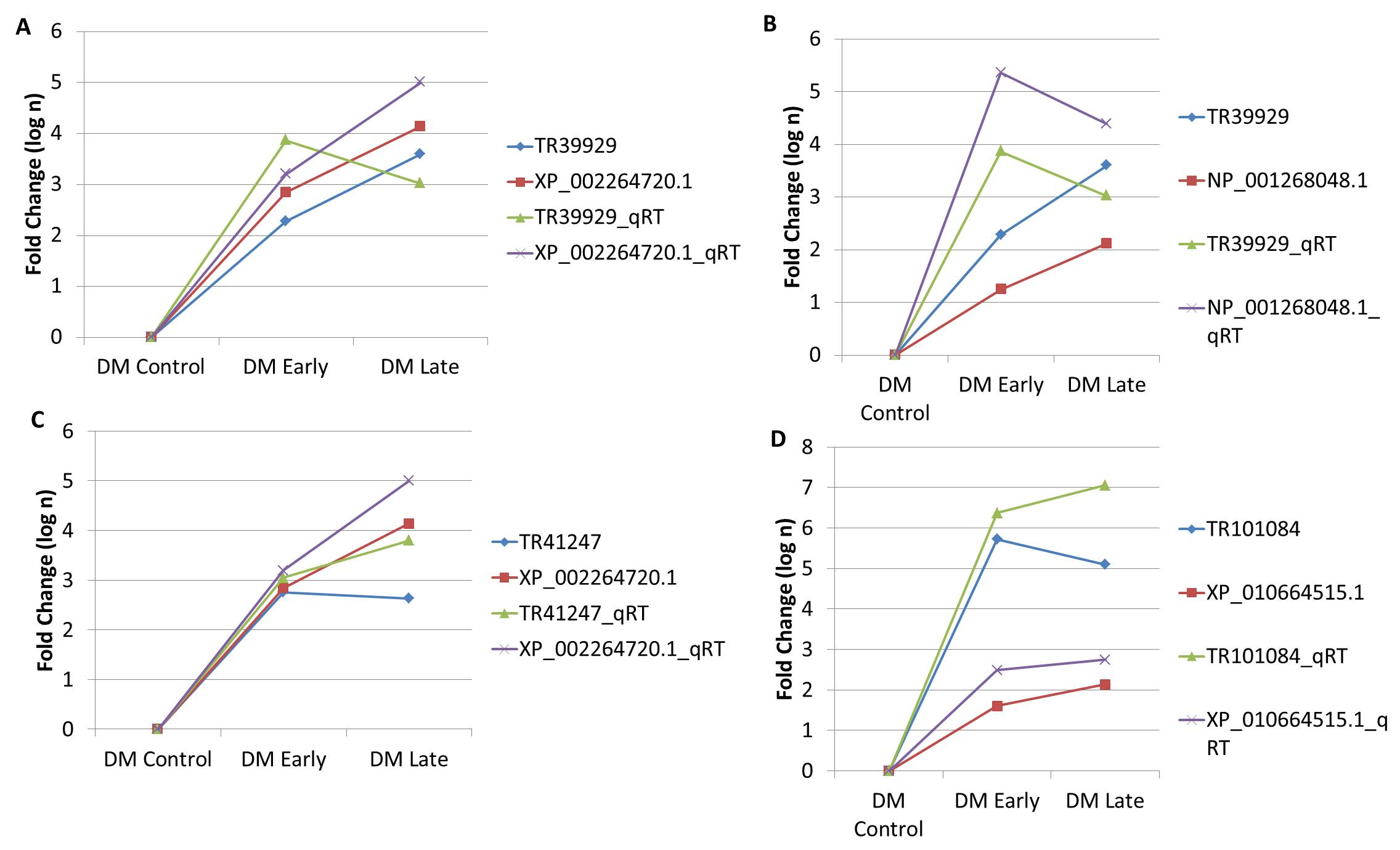


**Figure S14: Co-expression patterns of selected DM-responsive lncRNAs and corresponding protein coding sequences (CDS). (A-D) depict the co-expression patterns of 4 DM-responsive lncRNAs- CDS pairs.** The blue and red colors correspond to expression patterns observed by *in silico* differential expression analysis (FPKM values); while green and purple represent expression trends observed after qRT-PCR analysis. The names of the lncRNAs and NCBI reference sequence IDs of the CDS are provided in the color legends in each panel. The y-axis corresponds to the natural logarithm of the fold change values.
