# Supplementary figures and images for: Investigation of long non-coding RNAs as regulatory players of grapevine response to powdery and downy mildew infection
Source: BMC Plant Biol. 2021 Jun 8;21:265. doi: 10.1186/s12870-021-03059-6 (PMC8186045; doi:10.1186/s12870-021-03059-6)

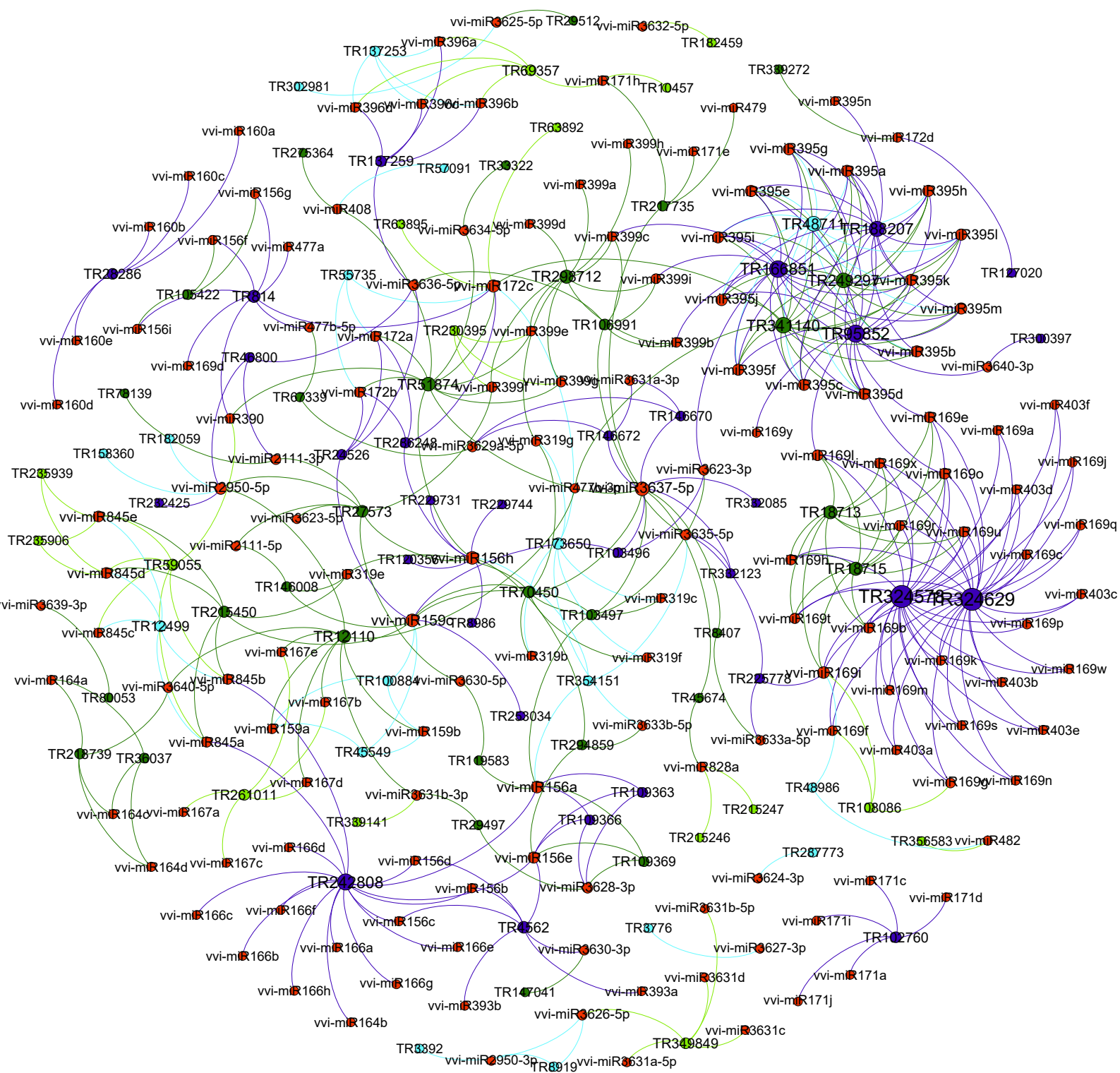

Supplement: Supplementary file 7 — Additional file 7: The interaction analyses of the PM- and DM-responsive lncRNAs with V. vinifera miRNAs to gain an overview of the interactome. [file 12870_2021_3059_MOESM7_ESM.pdf]
